# LncRNA *lncLy6C* induced by microbiota metabolite butyrate promotes differentiation of Ly6C^high^ to Ly6C^int/neg^ macrophages through *lncLy6C*/C/EBPβ/Nr4A1 axis

**DOI:** 10.1038/s41421-020-00211-8

**Published:** 2020-11-24

**Authors:** Yunhuan Gao, Jiang Zhou, Houbao Qi, Jianmei Wei, Yazheng Yang, Jianmei Yue, Xinqi Liu, Yuan Zhang, Rongcun Yang

**Affiliations:** 1 Department of Immunology, Nankai University School of Medicine, Nankai University, Tianjin 300071, China; 2 State Key Laboratory of Medicinal Chemical Biology, Nankai University, Tianjin 300071, China; 3 Key Laboratory of Bioactive Materials Ministry of Education, Nankai University, Tianjin 300071, China; 4 College of life Science, Nankai University, Tianjin 300071, China

**Keywords:** Autoimmunity, Transdifferentiation

## Abstract

Macrophages are mainly divided into two populations, which play a different role in physiological and pathological conditions. The differentiation of these cells may be regulated by transcription factors. However, it is unclear how to modulate these transcription factors to affect differentiation of these cells. Here, we found that *lncLy6C*, a novel ultraconserved lncRNA, promotes differentiation of Ly6C^high^ inflammatory monocytes into Ly6C^low/neg^ resident macrophages. We demonstrate that gut microbiota metabolites butyrate upregulates the expression of *lncLy6C*. *LncLy6C* deficient mice had markedly increased Ly6C^high^ pro-inflammatory monocytes and reduced Ly6C^neg^ resident macrophages. *LncLy6C* not only bound with transcription factor C/EBPβ but also bound with multiple lysine methyltransferases of H3K4me3 to specifically promote the enrichment of C/EBPβ and H3K4me3 marks on the promoter region of Nr4A1, which can promote Ly6C^high^ into Ly6C^neg^ macrophages. As a result, *lncLy6C* causes the upregulation of Nr4A1 to promote Ly6C^high^ inflammatory monocytes to differentiate into Ly6C^int/neg^ resident macrophages.

## Introduction

Monocytes/macrophages are involved in human diseases such as obesity, atherosclerosis, chronic obstructive pulmonary disease, lung fibrosis, lung cancer, and Alzheimer’s disease^1^. There are three classes of macrophages in humans, CD14^+^CD16^−^ (classical), CD14^+^ CD16^+^ (intermediate), and CD14^lo^ CD16^+^ (nonclassical) macrophages^2^, whereas in mice, two populations of macrophages Ly6C^hi^ CCR2^+^ CX_3_CR1^int^ and Ly6C^lo^ CCR2^−^ CX_3_CR1^hi^ have been described, representing classical and nonclassical monocytes respectively^3^. These cells were derived from monocyte/macrophage and DC progenitors (MDP)^4^, which may give rise to common monocyte progenitors (cMoP) committed to monocyte generation^5^.

In the steady-state, classical monocytes are maintained in the bone marrow (BM) and other extramedullary sites where they are available for immediate deployment to infected or injured tissues. Single-cell RNA sequencing also reveals that steady-state Ly6C^high^ monocytes possess neutrophil-like properties, including strong expression of granule proteins^6^. But nonclassical monocytes are recruited to noninflamed tissues, and characterized by their ability to patrol the resting vasculature, remove cell debris, and repair the endothelium^7–10^. Nonclassical macrophages are less proliferative than classical monocytes, but they remain in the circulation longer^10,11^. Most evidences indicate that nonclassical macrophages arise from classical monocytes in both mice and humans^9–11^. Multiple transcription factors such as runt-related transcription factor 3 are involved in the differentiation of macrophages^12–14^. CCAAT/enhancer binding protein β (C/EBPβ) plays a critical role in the differentiation of Ly6C^high^ macrophages^15,16^. Recent study also exhibits that transcription factor C/EBPβ-mediated Nr4A1 may promote the Ly6C^high^ into Ly6C ^low/neg^ macrophages^17^. However, it is unclear how to modulate C/EBPβ-mediated Nr4A1.

LncRNAs have diverse functions including the regulation of chromatin, gene expression, and signal transduction^18^. They play an important role in regulating differentiation and function of macrophages^19–21^. These lncRNAs can be either intergenic (between protein coding genes), intronic, natural antisense transcripts, or transcribed from divergent enhancers and promoters^22^. They may regulate gene expression in diverse biological processes through binding to chromatin-modifying factors and transcription factors^23^. Gut microbiota may regulate gut immune cells and systemic immune cells through multiple pathways^24^. It is not understood whether gut microbiota or their metabolites may regulate the expression of lncRNA(s) to affect expression of transcription factor(s) in macrophages. Here, we found that gut metabolites butyrate promotes the expression of LncRNA *lncLy6C*. We demonstrate that *lncLy6C* promotes the differentiation of Ly6C^high^ inflammatory macrophages into Ly6C^low/neg^ macrophages in peripheral blood. We also found that this lncRNA binds with transcription factor C/EBPβ and lysine methyltransferases of H3K4me3 to promote Nr4A1 expression.

## Results

### Butyrate promotes differentiation of the Ly6C^high^ into Ly6C^int/neg^ macrophages

**Fig. 1:**
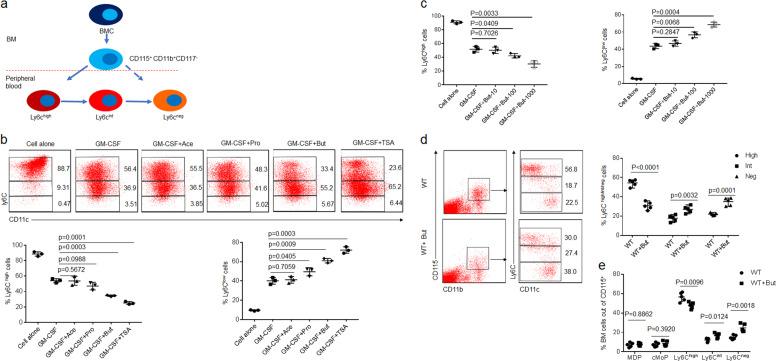
Butyrate promotes differentiation of Ly6C^int/neg^ macrophages. **a** Map of CD11b^+^CD115^+^CD117^−^ cell differentiation in peripheral blood. BMC bone marrow cells. **b** Flow cytometry of CD117^−^CD11b^+^CD115^+^Ly6C^+^ BM monocytes after exposed to ascetic acid (Ace, 200 μM), propionic acid (Pro, 200 μM), butyrate (But, 200 μM), and trichostatin A (TSA, 40 nM) for 4 days. Percentage of Ly6C^high^ and Ly6C^low^ cells was compared (lower). **c** Flow cytometry of CD117^−^CD11b^+^CD115^+^Ly6C^+^ BM monocytes after exposed to different concentration (0, 10, 100, 1000 μM) of butyrate (But). Percentage of Ly6C^high^ and Ly6C^low^ cells was compared. **d** Flow cytometry of CD11b^+^CD115^+^Ly6C^high^, CD11b^+^CD115^+^Ly6C^int^, and CD11b^+^CD115^+^Ly6C^neg^ cells in mice infused butyrate (But). Mice received sodium butyrate (150 mM/mouse) in the drinking water for 1 week (*n* = 6). Percentage of Ly6C^high^, Ly6C^int^, and Ly6C^neg^ macrophages were compared (*n* = 5). **e** Flow cytometry of BM cells in wt mice with (wt + But) or without (wt) the infusion. Two-sided Student’s *t* test in **d** and **e**; ANOVA in **b** and **c**; data for all panels are a representative from three experiments.

Monocytes (CD117^−^CD11b^+^CD115^+^Ly6C^+^) from BM may differentiate into CD11b^+^Ly6C^int^ and CD11b^**+**^Ly6C^neg^ cells in peripheral blood (Fig. 1a). To investigate effects of short-chain fat acids (SCFAs) on the differentiation of macrophages, we isolated CD117^−^CD11b^+^CD115^+^Ly6C^+^ BM monocytes. While butyrate was added into CD117^−^CD11b^+^CD115^+^Ly6C^+^ BM monocyte culture, we found that butyrate could promote the differentiation of Ly6C^high^ into Ly6C^low^ cells, whereas other SCFAs such as acetic acid or propionic acid did not do this (Fig. 1b; Supplementary Fig. S1). Trichostatin A (TSA), HDAC inhibitor^25^, also produced similar role in promoting the differentiation of Ly6C^high^ into Ly6C^low^ cells (Fig. 1b; Supplementary Fig. S1), suggesting that butyrate-mediated differentiation is through inhibiting HDAC. Furthermore, butyrate-mediated differentiation was dose dependent (Fig. 1c; Supplementary Fig. S2a). Meanwhile, butyrate also reduced the expression of TNFα, IL-6, IL-1β, and iNOS, whereas the expression of arginase-1, Fizz1, and Ym1 remarkably increased after exposed to butyrate (Supplementary Fig. S2b, c), consistent with other reports^26^. There exist two kinds of monocyte, including Ly6C^high^ and Ly6C^int/neg^ monocytes in peripheral blood^17^. We next determined whether butyrate also promoted differentiation of Ly6C^high^ to Ly6C^int/neg^ cells in peripheral blood. Since oral delivery of butyrate may target the small intestine and reach super-physiological concentrations in the periphery^27^, we directly infused butyrate into mice to observe its effects. Butyrate could markedly increase the proportion of Ly6C^neg^ monocytes, and meanwhile also reduce Ly6C^high^ monocytes in peripheral blood (Fig. 1d), whereas MDP and cMoP did not significantly change in BM (Fig. 1e; Supplementary Fig. S3). Thus, SCFA butyrate can directly affect the differentiation of Ly6C^high^ into Ly6C^int/neg^ macrophages.

### Butyrate induces LncRNA *lncLy6C* expression

**Fig. 2:**
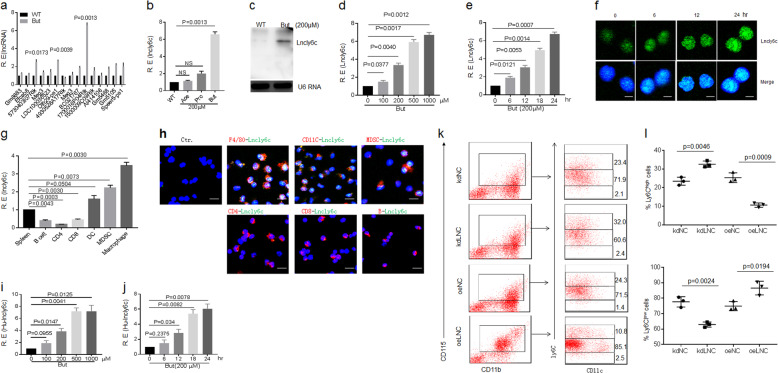
Butyrate promotes expression of *lncLy6C*. **a** QRT-PCR of lncRNAs in bone marrow-derived macrophages (BMDMs) after exposed to butyrate (200 μM). R. E relative expression. **b** QRT-PCR of *lncLy6C* in BMDMs after exposed to ascetic acid (200 μM), propionic acid (200 μM, and butyrate (200 μM). WT control vehicle, R. E relative expression. **c** Northern blot of *lncLy6C* in BMDMs after exposed to butyrate. WT control vehicle. **d** QRT-PCR of *lncLy6C* in BMDMs after exposed to different concentration of butyrate. R. E relative expression. **e** QRT-PCR of *lncLy6C* in BMDMs after exposed to butyrate (200 μM) at different time points. R. E relative expression. **f** FISH of *lncLy6C* in BMDMs after exposed to butyrate (200 μM) at different time points. Nuclei were stained with DAPI (blue); green, *lncLy6C*. Scale bar, 2.5 μM. **g** QRT-PCR of *lncLy6C* in spleen, B cell, CD4, CD8, dendritic cells (DC), myeloid-derived suppressive cells (MDSC), and macrophages sorted from spleen by flow cytometry. R. E relative expression. **h** FISH of *lncLy6C* and immunostaining of F4/80^+^, CD11c^+^, MDSC (ly6c^+^), CD4^+^, CD8^+^, and B (CD19^+^) cells in spleen. Nuclei were stained with DAPI (blue); green, *lncLy6C*. Scale bar, 40 μM. **i** QRT-PCR of *lncLy6C* in human CD14^+^ monocytes after exposed to different concentration of butyrate. R. E relative expression. **j** QRT-PCR of *lncLy6C* in human CD14^+^ monocytes after exposed to butyrate (200 μM) at different time points. R. E relative expression. **k** Flow cytometry of CD115^+^Ly6C^high^ and CD115^+^Ly6C^low^ cells in *lncLy6C* microRNA (kdLNC) or *lncLy6C* (oeLNC) lentivirus transfected BMCs after culturing for 4 days in the presence of GM-CSF. KdNC control microRNA lentiviruses, oeNC control lentiviruses. **l** Comparison f Ly6C^high^ and Ly6C^low^ cells in *lncLy6C* microRNA (kdLNC) or *lncLy6C* (oeLNC) lentivirus transfected BMCs and control microRNA (kdNC) or control lentivirus (oeNC) transfected BMCs, two-sided Student’s *t* test in **a**, **b**, **d**, **e**, **g**, **i**, **j**, and **l**; data for all panels are a representative from two to three experiments.

We next investigated how butyrate to induce differentiation of Ly6C^high^ to Ly6C^int/neg^ macrophages. LncRNAs play an important role in regulating macrophage differentiation and function^19–21^. We found that SCFA butyrate could induce a high level of the expression of lncRNA 1700016P04Rik (named as *lncLy6C*) in all detected lncRNAs, which were expressed by BM-derived macrophages (BMDMs)^28^ (Fig. 2a, b). The expression of *lncLy6C* could be furthermore confirmed using northern blot and fluorescence probe hybridization (FISH) (Fig. 2c, f). Butyrate-mediated *lncLy6C* expression was dose and time dependent (Fig. 2d–f). This lncRNA was only expressed in myeloid-derived cells such as macrophages, dendritic cells, and myeloid-derived suppressive cells (MDSCs), but not in CD4^+^ cells, CD8^+^ cells, and CD19^+^B cells (Fig. 2g, h). TSA^25^ also significantly promoted *lncLy6C* expression (Supplementary Fig. S4), suggesting that regulation of butyrate on the expression of *LncLy6C* is through inhibiting HDAC^29^. *LncLy6C* belongs to intergenic lncRNA (chromosome 6: 13413995–13510200), which was predominately localized to the nucleus and was without coding capacity (Fig. 2f; Supplementary Fig. S5a–g). This lncRNA was highly conserved between mouse and human (AC002463.1, named as hu*lncLy6C* (chromosome 7: 112447846–112728031) with 54.19% homology (Supplementary Fig. S6a). Hu*lncLy6C* could be detected in isolated human peripheral monocytes (Supplementary Fig. S6b). Butyrate could also regulate expression of Hu*lncLy6C* (Fig. 2i, j; Supplementary Fig. S7). Interestingly, gain and loss of function showed that this lncRNA could promote differentiation of Ly6C^high^ into Ly6C^int/neg^ macrophages. Silencing *lncLy6C* inhibited differentiation of CD11b^+^Ly6C^high^ into Ly6C^int/neg^ cells, which may be regulated by C/EBPβ transcription factor^17^, whereas transfection of *lncLy6C* could promote differentiation of CD11b^+^Ly6C^low^ cells from CD117^−^CD11b^+^CD115^+^Ly6C^+^ BM monocytes (Fig. 2k, l). Other lncRNAs such as Olfr29-PS1 did not have similar role (Supplementary Fig. S8). Furthermore, *lncLy6C* also has similar effects with butyrate on the expression of TNFα, IL-6, IL-1β, iNOS, arginase-1, FiZZ1, and Ym1 (Supplementary Fig. S9). Taken together, butyrate-mediated *lncLy6C* may induce differentiation of Ly6C^low^ macrophages.

### *LncLy6C* deficient mice have decreased Ly6C^neg^ macrophages

**Fig. 3:**
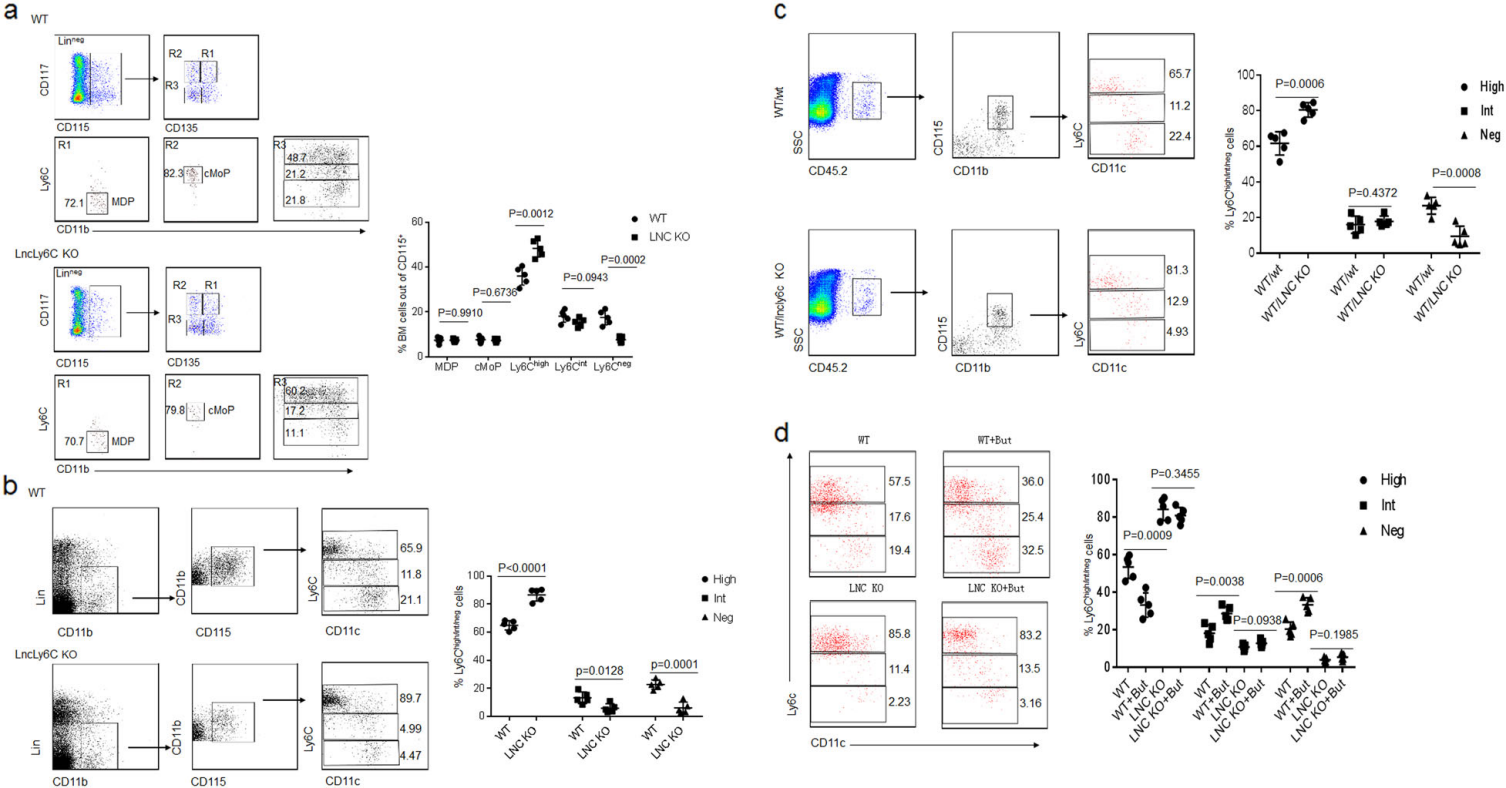
Reduced CD11b^+^ Ly6C^neg^ resident macrophages in the peripheral blood of *lncLy6C* deficient mice. **a** Flow cytometry of MDP, cMOP, and Ly6C^high^, Ly6C^int^ as well as Ly6C^neg^ macrophages in BM cells of wt and *lncLy6C* KO mice. Percentage of the cells was compared (right, *n* = 5). **b** Flow cytometry of Ly6C^high^, Ly6C^int^, and Ly6C^neg^ macrophages in peripheral blood of wt and *lncLy6C* KO mice. Percentage of Ly6C^high^, Ly6C^int^, and Ly6C^neg^ macrophages were compared (right, *n* = 5). **c** Flow cytometry of CD45.2^+^Ly6C^high^, Ly6C^int^, and Ly6C^neg^ in peripheral blood of CD45.1 mice, which were transplanted by CD117^−^CD11b^+^CD115^+^Ly6C^+^ BM monocytes from wt (WT/wt) or *lncLy6C* KO (WT/*lncLy6C* KO) mice after 48 h. Percentage of Ly6C^high^, Ly6C^int^, and Ly6C^neg^ macrophages were compared (*n* = 5). **d** Flow cytometry of Ly6C^high^, Ly6C^int^, and Ly6C^neg^ in peripheral blood of wt mice (WT + But) or *lncLy6C* KO (LNCKO + But) mice infused with butyrate. Mice received sodium butyrate (150 mM) in the drinking water for 1 week (*n* = 6). Percentage of Ly6C^high^, Ly6C^int^, and Ly6C^neg^ macrophages were compared with wt or *lncLy6C* ko (LNCKO) mice (*n* = 5). Two-sided Student’s *t* test; Data for all panels are a representative from three experiments.

To further investigate effects of *lncLy6C* on the differentiation of macrophages, we generated *lncLy6C* deficient mice. Increased Ly6C^high^ and markedly decreased Ly6C^int/neg^ macrophages could be found in the peripheral blood and BM of lycLy6C deficient mice, whereas MDP and cMoP did not significantly change in BM (Fig. 3a, b). Increased Ly6C^high^ cells were also found in the colonic tissues of lycLy6C deficient mice (Supplementary Fig. S10). To determine that the change of Ly6C^high^ and Ly6C^int/neg^ macrophages is indeed derived from *lncLy6C* deficient BM cell (BMC)s, we transplanted CD117^−^CD11b^+^CD115^+^Ly6C^+^ BM monocytes from *LncLy6C* KO or wild-type (wt) mice into CD45.1 mice. Cells were retrieved from recipient blood 1 week after transplanting BM cells and analyzed by flow cytometry. There had been markedly increased Ly6C^high^ and reduced Ly6C^int/neg^ CD45.2^+^ macrophages in *LncLy6C* KO transplanted mice, but not in mice transplanted by CD117^−^CD11b^+^CD115^+^Ly6C^+^ BM monocytes from wt mice (Fig. 3c), suggesting that *lncLy6C* in BM cells indeed play critical role in the differentiation of Ly6C^high^ into Ly6C^low^ macrophages. Unlike to those in wt mice, butyrate did not also cause increase Ly6C^low^ monocytes in peripheral blood of *LncLy6C* KO mice or DSS-treated *LncLy6C* KO mice (Fig. 3d; Supplementary Fig. S11). Taken together, *lncLy6C* deficiency inhibits the differentiation of Ly6C^high^ inflammatory macrophages into Ly6C^low^ resident macrophages.

### *LncLy6C* binds with C/EBPβ

**Fig. 4:**
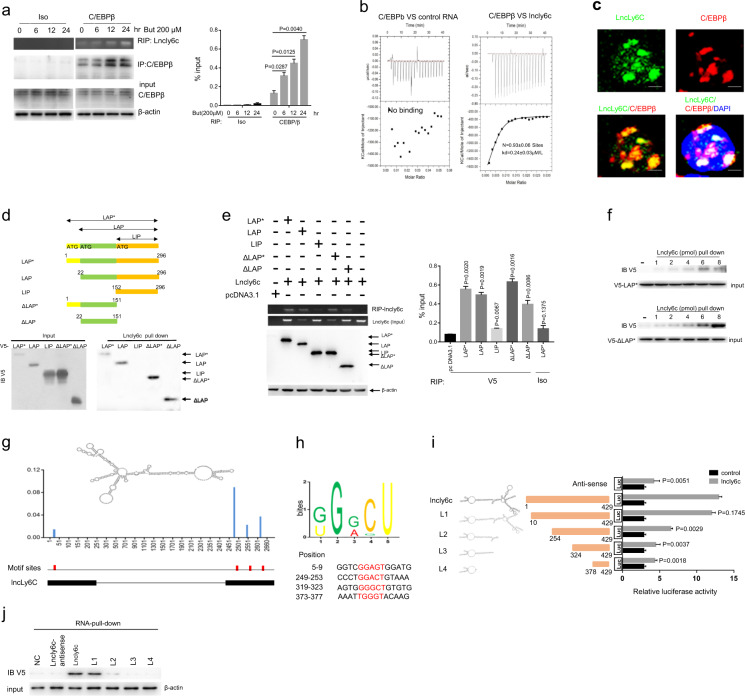
Binding of *lncLy6C* with CEBP/β. **a** RIP analyses of *lncLy6C* in BMDMs after exposed to butyric acid (200 μM) at different time points. **a** RIP was performed using anti-C/EBPβ antibody or control isotypic antibody (Iso) and then PCR for *lncLy6C*. % input (right) was analyzed. **b** ITC analysis of the binding of *lncLy6C* to CEBP/β. Control RNA, antisense RNA of *lncLy6C*. **c** Immunostaining and RNA-FISH of CEBP/β and *lncLy6C* in mouse BMDMs. Red, C/EBPβ; green, *lncLy6C*; blue, nuclei. Scale bar, 2.5 μM. **d** Pull-down analysis using biotinylated *lncLy6C* in V5-tagged C/EBPβ or V5-tagged C/EBPβ derivatives transfected HEK293T cells. C/EBPβ and its derivatives were cloned into pcDNA3.1/V5 to generate V5-tagged C/EBPβ and V5-tagged C/EBPβ derivatives, and then individually transfected into HEK293T cells. Biotinylated *lncLy6C* does not bind with C/EBPβ LIP. **e** RIP analyses in V5-tagged CEBP/β derivatives and *lncLy6C* cotransfected HEK293T cells. RIP was performed using anti-V5 antibody or control isotypic control (Iso) and then PCR for *lncLy6C*. % input (right) was analyzed. **f** Pull-down analysis using different concentrations of biotinylated *lncLy6C* in V5-tagged LAP* (V5-LAP*) (upper) or V5-tagged derivatives ΔLAP* (V5-ΔLAP*) (lower) transfected HEK293T cells. **g** Predicted structure of *lncLy6C* (RNA fold) with motifs (red). The iCLIP truncation track shows the positions of iCLIP cDNA truncations at lncRNA with peak height corresponding to the cDNA counts. The position of motifs sites was shown below the iCLIP cDNA truncation. **h** Sequence logo of C/EBPβ recognition motif generated by MEME analysis of lncRNA sequence read clusters (upper). Schematic of the corresponding regions of C/EBPβ binding motifs in *lncLy6C* (lower) and the motif portions are highlighted in red. **i** Luciferase analyses of HEK 293T cells cotransfected using different *lncLy6C* fragments with C/EBPβ LAP*. Different *lncLy6C* fragments were cloned into NF-κB report plasmids and then cotransfected with C/EBPβ LAP* into HEK293T cells. Luciferase activity was detected in cell lysates. **j** RNA pull down using biotinylated *lncLy6C* and fragments in V5-tagged C/EBPβ LAP* transfected HEK293T cells. NC no biotinylated *lncLy6C* and fragments. One-way ANOVA and Bonferroni’s multiple comparison test in **a** and **e**; two-sided Student’s *t* test in **i**.

LncRNA can affect the function of targeting molecules through multiple mechanisms such as binding with protein(s)^30^. Since C/EBPβ plays critical role in the survival and differentiation of Ly6C^high^ into Ly6C^low^ macrophages^15,17^. we hypothesized that *lncLy6C* could bind with C/EBPβ to cause the differentiation from Ly6C^high^ pro-inflammatory macrophages into Ly6C^int/neg^ resident macrophages. To investigate this, we first performed RNA immunoprecipitation (IP) analyses (RIP) using anti-C/EBPβ, we found that C/EBPβ could bind with *lncLy6C* (Fig. 4a). This binding was further confirmed using isothermal titration calorimetry (ITC) analyses (Fig. 4b). Fluorescence hybridization (FISH) also exhibited the binding of *lncLy6C* with C/EBPβ (Fig. 4c). C/EBPβ had three isoforms, C/EBPβ isoforms liver-enriched activator proteins (LAP* and LAP), which function as transcriptional activators, and C/EBPβ liver-enriched inhibitory protein (LIP), which lacks DNA transactivation domains but may form heterodimerized forms with other family members to control gene expression^31^. To investigate *lncLy6C* to bind with which isoform of C/EBPβ, we generated three different isoform of C/EBPβ, and meanwhile also generated different derivatives with tagged V5 (Fig. 4d), we found that only N-terminal region (aa 22–151) of LAP* was necessary for binding with *lncLy6C* through *lncLy6C* pull down and RIP analyses (Fig. 4d, e). Using different concentration of *lncLy6C* to further examine binding with C/EBPβ (LAP*) results showed that the binding with N-terminal region was *lncLy6C* dose dependent (Fig. 4f). Taken together, data suggest that *lncLy6C* can bind to N-terminal region (aa 22–151) of C/EBPβ (LAP* and LAP). We also determined the functional motif in *lncLy6C*. To found the potential motif in *lncLy6C*, which is bound by C/EBPβ, we first got lncRNA by RIP using anti-C/EBPβ antibody, and then digested by RNA enzymes to establish cDNA library after amplification. By MEME algorithm analyses, we found potential binding motif(s) in *lncLy6C*, which may interact with C/EBPβ (Fig. 4g, h). Next, we examined which motif could bind with C/EBPβ. We found that a fragment containing motif (5′-249-GGACT-253 3′) in N terminal of *lncLy6C* was involved in the binding of *lncLy6C* to C/EBPβ (Fig. 4i). The pull down using the fragments containing different motifs also confirmed this motif, which could bind with C/EBPβ (Fig. 4j). Thus, our data indicate that *lncLy6C* may bind with C/EBPβ through *lncLy6C* motif (5′-249-GGACT-253 3′).

### Methylation of *lncLy6C* 5′ adenosine^251^ determines binding of *lncLy6C* to C/EBPβ

**Fig. 5:**
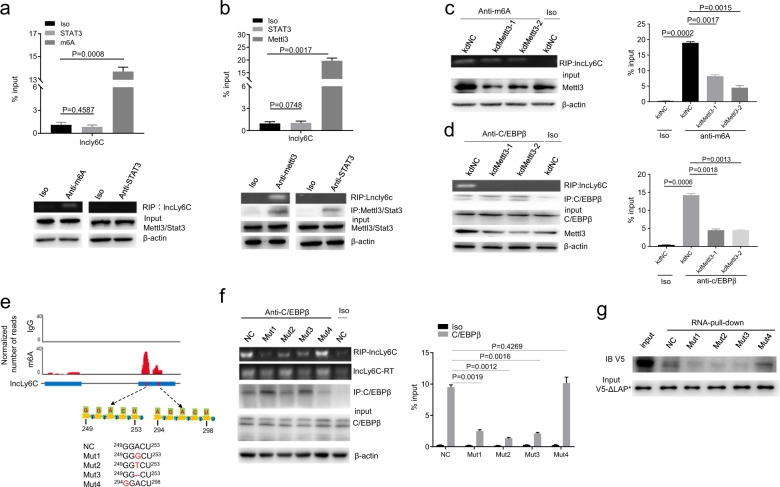
Binding of *lncLy6C* to C/EBPβ depends on m6A methylation. **a** RIP analyses in BMDMs after exposed to butyrate. RIP was performed using anti-m6A antibody and then PCR for *lncLy6C*. Iso, isotypic antibody; anti-STAT3 (STAT3), negative control. % input was compared. **b** RIP analyses in BMDMs after exposed to butyrate (200 μM). RIP was performed using anti-Mettl3 antibody and then PCR for *lncLy6C*. Iso, isotypic antibody; anti-STAT3 (STAT3), negative control. % input was compared. **c** RIP analyses in mettl3 siRNA transfected BMDMs. RIP was performed using anti-m6A antibody and then PCR for *lncLy6C*. kdNC, siRNA control; KdMettl3-1 and KdMettl3-2, different mettl3 siRNA; Iso, isotypic antibody. % input (lower) was compared. **d** RIP analyses of mettl3 siRNA transfected BMDMs. RIP was performed using anti-C/EBPβ antibody and then PCR for *lncLy6C*. kdNC, siRNA control; KdMettl3-1 and KdMettl3-2, different mettl3 siRNAs; Iso, isotypic antibody. % input (lower) was compared. **e** m6A RIP-seq of *lncLy6C* and sequence logo of *lncLy6C* with m6A methylation motifs. m6A peaks are enriched in the *lncLy6C* gene. The iCLIP truncations track shows the positions of iCLIP cDNA truncations at lncRNA with peak height corresponding to cDNA counts. Schematic of the corresponding regions of CEBP/β binding motifs in lncRNA (low) and the mutant base in motif portions are labeled in red. **f** RIP-PCR analysis in HEK293T cells cotransfected with C/EBPβ expression vector and different lncRNA mutants. RIP was performed using anti-CEBP/β and then PCR for lncRNA. % input of *lncLy6C* was analyzed (lower). NC, full length *lncLy6C*. **g** RNA pull down using biotinylated *lncLy6C* (NC) or *lncLy6C* mutants in V5-tagged C/EBPβ LAP* transfected HEK293T cells. One-way ANOVA and Bonferroni’s multiple comparison test in **b**. Two-sided Student’s *t* test in **c**. ANOVA and Bonferroni’s multiple comparison test in **a**–**d** and **f**.

N6-methyladenosine (m6A) is the most abundant internal modification in eukaryotic messenger RNAs (mRNAs) and lncRNAs. It plays an important role in the function of RNA^32^. m6A can be catalyzed by methyltransferase like (METTL) 3, METTL14, and Wilms tumor 1-associated protein (WTAP)^33^. Using anti-m6a or anti-METTL3 antibody, we found that *lncLy6C* possessed m6A (Fig. 5a, b). Thus, we next examined whether this methylation also play a role in *lncLy6C*. Silencing METTL3 affected the binding of *lncLy6C* with C/EBPβ by RIP using anti-m6A or anti-C/EBPβ (Fig. 5c, d). We next analyzed the position of potential m6A site(s) through sequencing after UV-RIP. There had multiple points, which could be methylated (Fig. 5e). Importantly, adenosine^251^ in 5′-249-GGACT-253 3′ motif, which determines the binding of *lncLy6C* to C/EBPβ, could be methylated (Fig. 5e). The transversion, replacement, and deletion of adenosine^251^ interrupted the binding of *lncLy6C* to C/EBPβ (Fig. 5f). RIP and pull down also did not reveal the interaction of these mutants with C/EBPβ (Fig. 5g). Thus, N6-methylation on 5′ adenosine^251^ determines binding of *lncLy6C* to C/EBPβ.

### *LncLy6C* binds with lysine methyltransferases of H3K4me3

**Fig. 6:**
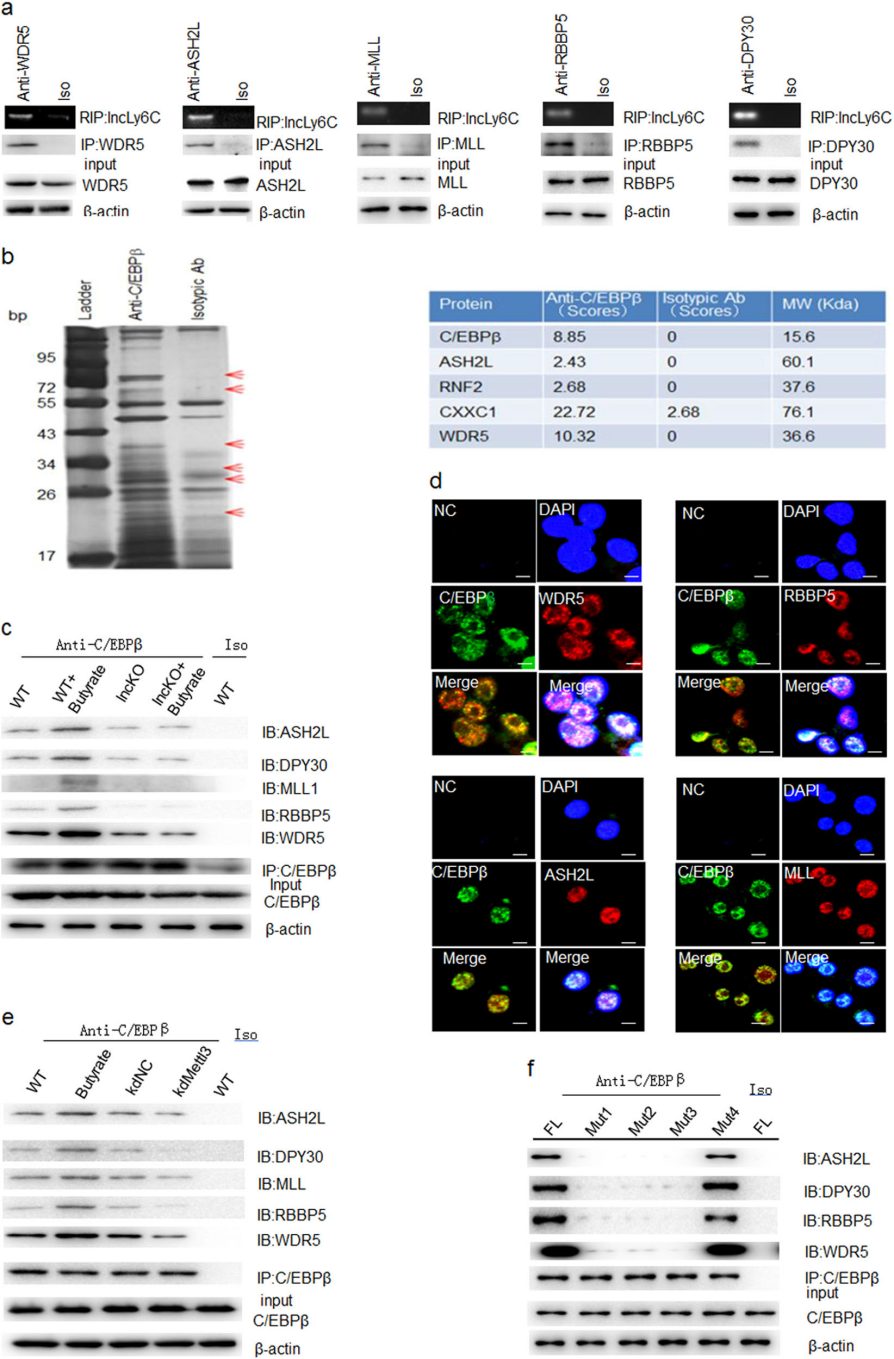
*LncLy6C* binds with lysine methyltransferases of H3K4me3. **a** RIP analyses of BMDMs after exposed to butyrate. RIP was performed using different antibodies including anti-WDR5, -ASH2L, -MLL, -RBBP5, or -DPY30 and then PCR for *lncLy6C*. **b** Immunoprecipitation in BMDMs using anti-C/EBPβ antibody. ASH2L, RNF2, CXXC1, WDR5, etc. were found in IP–MASS analyses. **c** Immunoprecipitation in BMDMs using anti-C/EBPβ. ASH2L, DPY30, MLL, RBBP5, and WDR5 were detected in BMDM (WT), butyrate-treated BMDMs (WT + butyrate), BMDMs from *lncLy6C* KO (lncKO) or butyrate-treated *lncLy6C* KO BMDMs (Lnc KO + butyrate). Iso, antibody control. **d** Immunostaining of C/EBPβ with lysine methyltransferases (WDR5, RBBP5, ASH2L, or MLL) of H3K4me3 in BMDMs after exposed to butyrate (200 μM). Scale bar, 2.5 μM. **e** Immunoprecipitation in BMDMs using anti-C/EBPβ. WT, BMDM; butyric acid, butyrate-treated BMDM; kdNC, control siRNA transfected BMDMs; kdMettl3, mettl3 siRNA transfected BMDMs. Iso, isotypic antibody control. **f** Coimmunoprecipitation of C/EBPβ with ASH2L, DPY30, RBBP5, or WDR5 in cotransfected HEK293T cells using different *lncLy6C* fragments with mutated m6a methylation sites (Mut1, Mut2, Mut3, and Mut4 in Fig. 5e) and C/EBPβ as well as ASH2L, DPY30, RBBP5, or WDR5. FL full length *lncLy6C*; Iso isotypic control.

Accumulation of H3K4me3 marks on multiple immune gene promoters may underlie robust transcriptional responses. But, the mechanism how these epigenetic marks accumulate at a specific immune gene locus has been poorly understood^34^. One possibility is directly recruitment of lysine methyltransferase complexes by lncRNA. Several reports have indicated critical roles of lncRNAs in promoting enrichment of H3K4me3 marks^34–36^. The enrichment of H3K4me3 mark depends on “core complexes” such as WD repeat containing protein 5 (WDR5), absent, small, or homeotic 2-like protein (ASH2L), mixed-lineage leukemia, and retinoblastoma-binding protein 5 (RBBP5), which catalyze H3K4me3 methylation^37^. Thus, we examined whether *lncLy6C* could also bind with these lysine methyltransferases. Results showed that *LncLy6C* could bind with the components of H3K4me3 methylation complexes, including WDR5, ASH2L, MLL, RBBP5, and DPY30 (Fig. 6a). Since *lncLy6C* may bind with C/EBPβ, we also investigated whether C/EBPβ could interact with these components. IP–mass spectrometric analyses using anti-C/EBPβ antibody exhibited that C/EBPβ could bind with WDR5 and ASH2L (Fig. 6b; Supplementary Table S1). Further studies showed that C/EBPβ could not only bind with WDR5 and ASH2L but also DPY30, MLL1, and RBPP5 (Fig. 6c, d). H3K4 can be mono-, di-, or trimethylated (H3K4me1, me2, or me3, respectively) by different enzymes^37,38^. However, C/EBPβ could not bind with enzymes such as SET7/9, which promotes mono- or dimethylation of lysine^39^. Butyrate, which can induce the expression of *lncLy6C*, also promoted these binding, whereas it did not affect their binding in *lncLy6C* deficient macrophages (Fig. 6c, d). The binding of C/EBPβ with lysine methyltransferases was also affected by m6A enzyme. Silencing mettl3 could decrease their binding (Fig. 6e). The mutation of 5′ adenosine^251^ also interrupted the binding of *lncLy6C* with the components of core complexes (Fig. 6f). Thus, we demonstrate that *lncLy6C* not only binds with C/EBPβ but also binds with lysine methyltransferases of H3K4me3.

### *LncLy6C* promotes enrichment of C/EBPβ and H3K4me3 mark on the promoter region of Nr4A1

**Fig. 7:**
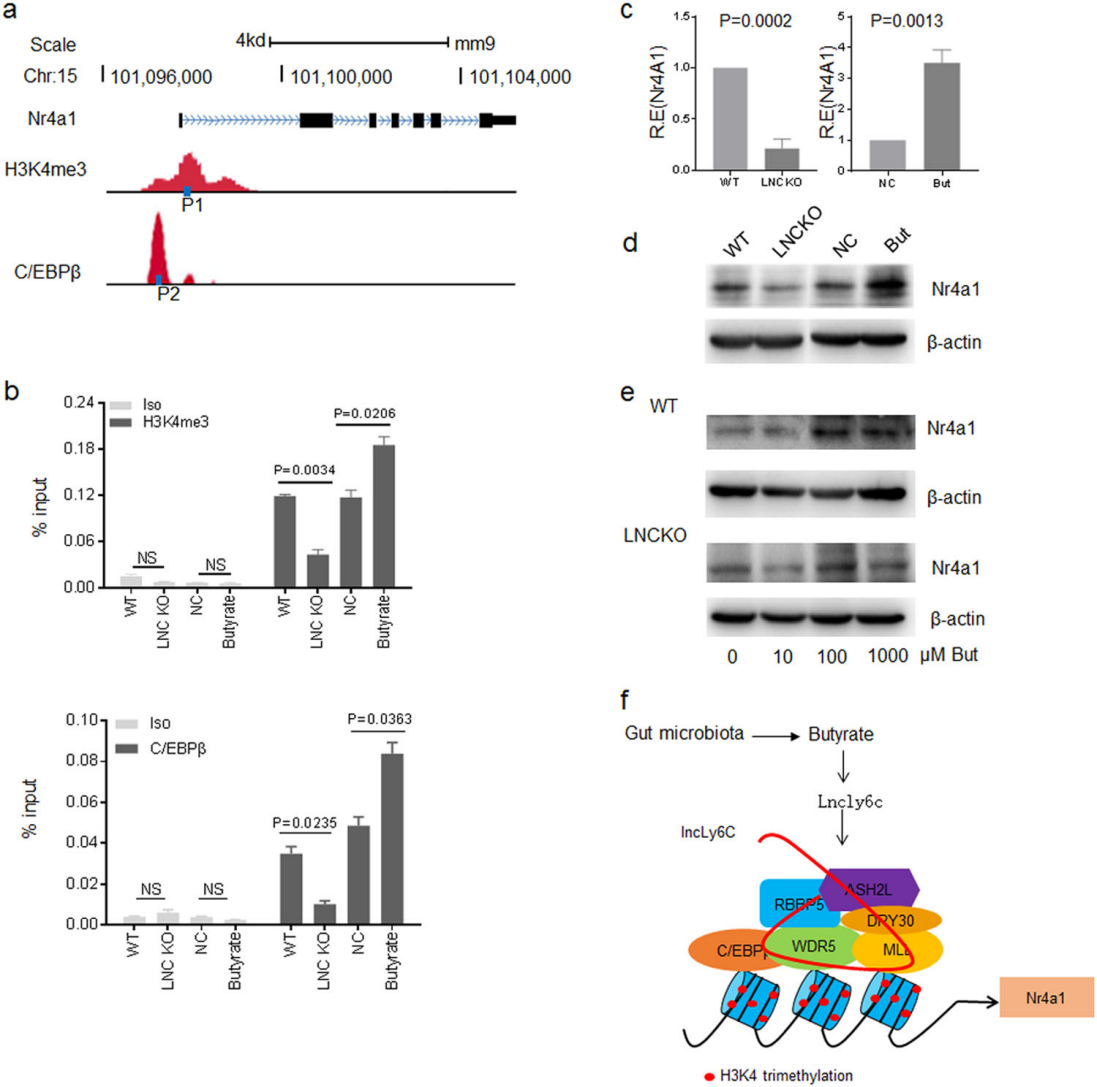
*LncLy6C* promotes enrichment of C/EBPβ and H3K4me3 on the promoter region of Nr4A1. **a** Map of H3K4me3 marks, C/EBPβ binding sites, P1 and P2 sites of CHIP-PCR on the promoter region of Nr4A1. **b** CHIP-PCR in BMDM of wt and *LncLy6C* KO (LNC KO), control BMDM (NC), and butyrate-treated BMDMs (butyrate). % input was compared. Iso isotypic antibody. **c** QRT-PCR of Nr4A1 in BMDM of wt and *LncLy6C* KO (LNC KO) (left) or control BMDM (NC) and butyrate-treated BMDMs (Buty) (right). **d** Immunoblotting of Nr4A1 in BMDM of wt and *LncLy6C* KO (LNC KO) or control BMDM (NC) and butyrate-treated BMDMs (butyrate). **e** Immunoblotting of Nr4A1 in the BMDM of wt and *LncLy6C* KO (LNC KO) after exposed to different concentration of butyrate. **f** Map of butyrate-mediated *lncLy6C* to regulate the expression of Nr4A1. Butyrate-induced *LncLy6C* may promote the expression of Nr4A1 through binding with C/EBP and lysine methyltransferases of H3K4me3. One-way ANOVA and Bonferroni’s multiple comparison test in **b**. Two-sided Student’s *t* test in **c**.

*LncLy6C* not only binds with C/EBPβ but also binds with core complex components of H3K4me3, suggesting a mechanism that transcription factor C/EBPβ can interact with distinct histone methyltransferase complexes under the assistance of *lncLy6C* to induce the expression of a specific immune gene. Nr4A1 play an important role in Ly6C^high^ inflammatory macrophages to Ly6C^int/neg^ resident macrophages^16,17^. Genome browser image from public repository showed that there had both a H3K4 methylation marks and C/EBPβ binding sites on the promoter region of Nr4A1 (Fig. 7a). We investigated whether *lncLy6C* deficiency affects the enrichment of H3K4me3 and C/EBPβ in the promoter region of Nr4A1 to interrupt the expression of Nr4A1. Indeed, chromatin IP (CHIP)-PCR exhibited that *lncLy6C* deficient macrophages had reduced enrichment of C/EBPβ and H3K4me3 marks on the promoter region of Nr4A1 (Fig. 7b). But butyrate, which can induce *lncLy6C*, could cause the enrichment of C/EBPβ and H3K4me3 (Fig. 7b). Finally, *lncLy6C* deficient macrophages exhibited less expression of Nr4A1, whereas butyrate promoted Nr4A1 expression (Fig. 7c, d). Furthermore, butyrate induced the expression of Nr4A1 only in wt mice derived BMDMs but not in *lncLy6C* deficient mice derived BMDMs (Fig. 7e). Thus, our results demonstrate that butyrate-mediated *lncLy6C* may promote the enrichment of C/EBPβ and H3K4me3 to specifically induce the expression of Nr4A1 (Fig. 7f).

## Discussion

Here, we found that gut microbiota derived butyrate promotes the expression of LncRNA *lncLy6C*. We demonstrate that *lncLy6C* binds with transcription factors C/EBPβ and multiple lysine methyltransferases to promote the expression of Nr4A1. We also found that *lncLy6C* deficient mice have remarkably decreased Ly6C^neg^ macrophage population, and demonstrate that differentiation of Ly6C^neg^ macrophages is dependent on *lncLy6C* in BM cells. Thus, our results indicate that butyrate-induced *lncLy6C* promotes the differentiation of Ly6C^high^ inflammatory macrophages into Ly6C^int/neg^ resident macrophages through modulating C/EBPβ-mediated Nr4A1.

Butyrate is one class of short-chain fatty acid, the main metabolites produced by bacterial fermentation of dietary fiber in the gastrointestinal tract^40^. It is widely recognized to be capable of inhibiting the expression of pro-inflammatory cytokines^41^ and modulate intestinal macrophage function via histone deacetylase inhibition, thereby contributing to homeostasis in the intestines^42^. Interestingly, despite the low concentration in the periphery, butyrate may affect peripheral organs^27^ and peripheral immune system^43–45^. Butyrate may regulate the differentiation, recruitment, and activation of neutrophils, dendritic cells, macrophages and monocytes, and T cells^46^. In vitro culture, butyrate facilitates M2 macrophage polarization and function^26^. It has anti-inflammatory effects on LPS-mediated M1 macrophage via reducing production of pro-inflammatory mediators such as NO and IL-6^42^. We here demonstrate that butyrate may upregulate the expression of *lncLy6C*, which may promote Ly6C^high^ inflammatory macrophages into Ly6C^int/neg^ resident macrophages. Butyrate-induced Ly6C^high^ to Ly6C^int/neg^ macrophages transformation has an important physiological and clinical significance in preventing the occurrence and development of colitis. In addition, since *lncly6c* is clearly induced by butyrate in macrophages, it is interesting to investigate the role(s) of *lncly6c* in macrophages.

We demonstrate that *lncLy6C* binds with C/EBPβ LAP* isoform to induce Ly6C^high^ pro-inflammatory macrophages into Ly6C^int/neg^ resident macrophages. C/EBPβ isoforms liver-enriched activator proteins LAP* and LAP function are acted as transcriptional activators, whereas C/EBPβ LIP lacks DNA transactivation domains but LIP may form heterodimerized form with other family members to control the gene expression^31^. Other also found that C/EBPβ may regulate differentiation of macrophages^15,16^ and that C/EBPβ is required for survival of Ly6C monocytes^15,17,47^. C/EBPβ also is necessary for the immunosuppressive program in both tumor-induced and BM-derived MDSCs and play a critical role in regulating the expression of immune suppressive genes^48,49^. *lncLy6C* not only bind with LAP* isoform but also bind with multiple components of enzyme complexes, which may promote accumulation of H3K4me3 epigenetic marks on the promoter region of Nr4A1. Enrichment of H3K4me3 is positively correlated with transcriptional activity^50^. The lysine methyltransferase “core complexes,” which may induce the methylation of H3K4me3, include WDR5, RBBP5, ASH2L, DPY30 called WRAD, and MLL^51^. The binding of *lncLy6C* with these components of “core complexes” may strengthen the formation of the complexes to promote the expression of gene. Indeed, our data showed the binding of *lncLy6C* promote the both accumulation of C/EBPβ and enzyme components on the promote region of Nr4A1 to induce the expression of Nr4A1. WDR5 may also bind with other lncRNAs to regulate the gene expression such as that WDR5 binds with lncRNA HOTTIP RNA to drive histone H3 lysine 4 trimethylation and gene transcription^36^. HoxBlinc is encoded by a gene in the HOXB cluster. Similar to HOTTIP, the knockdown or knockout of HoxBlinc results in reduced expression of HOXB genes^52^. Other also found that Fendrr, another lncRNA, may also interact with methyltransferase complexes^53^.

Histone modifications may be modulated by chromatin-modifying enzymes including chromatin remodeling complexes such as histone methyltransferases. Furthermore, histone modifications also recruit nonhistone proteins to further modify chromatin such as the binding of C/EBPβ to CBP/p300^54^ and physical interactions of C/EBPβ with the MLL3/MLL4 complexes^55^. Our results reveal that these modifying processes may include lncRNAs such as *lncLy6C*, which not only binds with C/EBPβ but also bind with multiple histone methyltransferases. Several studies also show that lncRNAs may tether protein-interacting partners near target genes to regulate their transcription^56,57^. Thus, our study suggests a model for a specific gene expression, that transcription factor C/EBPβ can interact with distinct histone methyltransferase complexes under the assistance of *lncLy6C* to promote the expression of Nr4A1.

## Materials and methods

### Mice and cell lines

*LncLy6C* (1700016P04Rik) deficient mice on a C57BL/6J background were generated by the Model Animal Research Center of Nanjing University (Nanjing, Jiangsu, China) using CRISPR-Cas9 system. Cas9 mRNA and sgRNA were coinjected into zygotes, sgRNA direct Cas9 endonuclease cleavage in upstream of exon 1 of *LncLy6C* and downstream of exon 2 of *LncLy6C*, and create a double-strand break. Such breaks were repaired by nonhomologous end joining, and resulted in deletion of 1700016P04Rik gene. Generated *lncLy6C* deficient mice were cultured and maintained in a specific pathogen-free (SPF) condition. C57BL/6 and B6.SJL-CD45a(Ly5a) (CD45.1) mice were also purchased from the Model Animal Research Center of Nanjing University (Nanjing, Jiangsu, China) and maintained in a SPF condition. All animal experiments were carried out in accordance with Nankai University Guide for the Care and Use of Laboratory Animals. Human embryonic kidney cell line HEK 293T cells were obtained from the American Type Culture Collection.

### In vitro culture

For generation of BMDMs, BMDMs were generated form BMCs in RPMI1640 medium with 10% FCS in the presence of M-CSF (30 ng/ml) for 4 days.

For the differentiation of CD117^−^CD11b^+^CD115^+^Ly6C^+^ cells, CD117^−^CD11b^+^CD115^+^Ly6C^+^ cells were sorted using flow cytometry, and then cultured in the RPMI1640 medium with 10 ng/ml GM-CSF with or without butyrate (200 μM) at the indicated concentration for 4 days.

### Chimeric mouse model

For chimeric mouse model, CD117^−^CD11b^+^CD115^+^Ly6C^+^ cells sorted from the BM cells of wt or *lncLy6C* KO mice (CD45.2) were injected to CD45.1 mice in tail vein (1 × 10^7^/mouse). After 1 week, the CD45.2^+^CD115^+^CD11b^+^Ly6C^+^ and CD45.2^+^CD115^+^CD11b^+^Ly6C^−^ cells were analyzed using flow cytometry.

Flow cytometry Cells were collected and rinsed twice with ice-cold PBS, incubated with FITC-, PE-, percy5.5-, or APC-labeled antibodies for 30 min in PBS with 1% FBS. After washed twice, cells were resuspended in PBS and analyzed using a FACScan flow cytometer. Antibodies used in flow cytometry were listed in Supplementary Table S2.

### 5′- and 3′-RACE for lncLy6C

First choice RNA-ligation mediated RACE kit (Ambion) was used to obtain full sequence of *lncLy6C*. RT-PCR using a *lncLy6C* specific primer and a primer binding to the ligated RNA adapter was performed to amplify the ligated lncLy6C followed by TOPO TA cloning and sequencing to determine the 5′ and 3′ end sequences of the lncRNA. The *lncLy6C* specific primers are listed in Supplementary Table S2.

### SiRNAs, lentiviruses, and plasmid construction

SiRNAs were purchased from Riobio (Guangzhou, China). SiRNA sequences for Mettl3 were listed in Supplementary Table S2. *LncLy6C* shRNA targets were chosen from the target sequences produced by BLOCK-iT™ RNAi Designer (Invitrogen) and/or by i-Score Designer. The shRNA constructs were generated using pGreenPuro™ shRNA Cloning and Expression Lentivector Kit (System Biosciences Inc.) according to the manual. The control shNC is the luciferase control shRNA from the kit. For packaging of lentivirus particles, the shRNA lentivector together with pMD2G and psPAX2 packaging plasmids were cotransfected into 293T cells. For preparation of plasmids, the sequences of C/EBPβ, including LAP*, LAP, LIP, ΔLAP*, ΔLAP, WDR5,ASH2L, DPY30, RBBP5, and *lncLy6C*, were amplified using PCR methods (primer pairs used are described in Supplementary Table S2). The PCR products were cloned into the pcDNA™ 3.1/V5-His TOPO^®^ TA plasmid (Invitrogen). After sequencing, plasmid constructions were used to transfect HEK 293T.

### RNA extraction and quantitative real-time PCR (qRT-PCR)

Total RNA was extracted from the cells using TRIzol reagent (Invitrogen). First-strand cDNA was generated from total RNA using oligo-dT/random primer mix and reverse transcriptase (Invitrogen Corp). qRT-PCR was conducted using QuantiTect SYBR Green PCR Master Mix (Qiagen) and specific primers in an ABI Prism 7000 analyzer (Applied Biosystems). GAPDH mRNA expression was detected in each experimental sample as an endogenous control. The fold changes were calculated using the ∆∆Ct method according to the manufacturer’s instructions (Applied Biosystems). All the reactions were run in triplicate.

### Northern blot

For northern blot, harvested total RNAs were run on 1% agarose formaldehyde gel, and then transferred to a Hybond nylon membrane using the Trans-Blot SD semi-dry electrophoretic transfer (Bio-Rad). Membrane was prehybridized for 1 h at 42 °C and incubated with the probe overnight at the same temperature. After washing, membrane was blocked and incubated with digoxin antibody conjugated with horseradish peroxidase (HRP). The primers used for the DIG-labeling probe preparation are listed in Supplementary Table S2.

### Western blot

For western blot analyses, our previous method^58^ was used in this study. Briefly, cells were harvested at the indicated times and rinsed twice with ice-cold PBS. Cell extracts were prepared with lysis buffer and centrifuged at 13,000× *g* for 10 min at 4 °C. Protein samples were electrophoresed using 12% polyacrylamide gels and transferred to PVDF membranes. After the membranes were blocked with 5% skim milk powder for 1 h at room temperature, they were incubated with first antibody in TBST overnight at 4 °C. Secondary antibodies with HRP (1:10,000) were labeled according to our previous method. The signals were checked by autoradiography film when HRP substrate was added to the membranes.

### Immunostaining and RNA-FISH

Immunostaining and RNA Fluorescence in situ hybridization (RNA-FISH) were performed according to our reported protocol^59^. Briefly, cells were first slicked on sterile and 0.01% poly-lysine-treated slides in the bottom of a six-well tissue culture dish. After that, the slides were processed sequentially with ice-cold CSK buffer, CSK + 0.4% Triton X-100 buffer and CSK buffer for 30 s for cell membrane perforation. The slides were then treated with 4% PFA for 10 min and cold 70% ethanol three times for cells fixation. After rinsed three times with ice-cold PBS, the slides were blocked in pre-warmed 5% goat serum for 30 min at 37 °C. Then, the slides were incubated with primary antibody at 37 °C for 1 h, washed three times with 1× PBS/0.2% Tween-20 for 3 min on a rocker, and then incubated with secondary antibody at 37 °C for 30 min. The slides were dehydrated by moving them through a room temperature ethanol series (85%, 95%, and 100% ethanol) for 2 min each, and air-dried at room temperature for 15 min and hybridized using the indicated probes overnight at 37 °C in a humid chamber. After washing with 2× SSC/50% formamide, 2× SSC, and 1× SSC each for three times, DAPI dye was added. Finally, the slides were sealed, and then observed using confocal microscope. The sequences of *lncLy6C* probe and control probe were listed in Supplementary Table S2.

### RNA immunoprecipitation

RIP was performed according to previously reported protocol^28^. Briefly, the cells were harvested, washed, added ice-cold IP lysis buffer (Thermo Scientific Pierce) containing 0.5% ribonuclease inhibitor (Invitrogen), and incubated on ice for 5 min with periodic mixing. Then, the lysates were transferred into a microcentrifuge tube and centrifuged at 13,000× *g* for 10 min to pellet the cell debris at 4 °C, and the supernatants were transferred into a new tube, and protein G agarose was added and incubated for 1 h at 4 °C with rotation for preclearing. The immunoprecipitating antibody was added and incubated overnight at 4 °C with rotation. Protein G agarose was pelleted by brief centrifugation (3000× *g* for 1 min) and then washed sequentially with IP lysis buffer (containing 0.5% ribonuclease inhibitor). Finally, RNA was extracted from protein/RNA complexes on the beads using TRIzol reagent and dissolve in DEPC-water and quantified by quantitative PCR (qPCR).

### Isothermal titration calorimetry

Calorimetric experiments were conducted at 25 °C with a MicroCal iTC200 instrument. C/EBPβ protein was dialyzed against the titration buffer containing 20 mM Tris-HCL, pH 7.4, 150 mM NaCl, and 2 mM MgCl_2_. Lyophilized RNA samples were prepared in the titration buffer, renatured at 95 °C for 2 min, 4 °C for 2 min, and 25 °C for 20 min, and then diluted to be required concentration for ITC. Acquired calorimetric titration data were analyzed using software origin 7.0 based on the “One Set of Sites” fitting model.

### RNA-protein pull-down analyses

RNA-protein pull-down analyses were performed using Pierce™ Magnetic RNA-Protein Pull-Down Kit. Transfected and induced cells were harvested and cell lysates were prepared using IP lysis buffers (Thermo Scientific Pierce). LncLy6C and its mutants were transcribed (NEB, Manual HiScribe T7 In Vitro Transcription Kit) and labeled using RNA 3′ Desthiobiotinylation Kit (Thermo Scientific Pierce) in vitro. Fifty microliters of beads and fifty picomoles of labeled RNA were added into RNA capture buffer, and incubated for 30 min at room temperature with agitation to binding of labeled lncLy6C to streptavidin magnetic beads. After washing beads with an equal volume of 20 mM Tris (pH 7.5), 100 µl of protein–RNA binding buffer was added into the beads and mixed well. One hundred microliters of master mix of RNA-protein binding reaction was added to the RNA-bound beads, mixed by pipetting and then incubated 60 min at 4 °C with rotation to binding of RNA-binding proteins to RNA. After washing beads with 100 μl wash buffer for twice, 50 μl of elution buffer was added and incubated 30 min at 37 °C with agitation. The samples were analyzed on a gel.

### UV-RIP

The cells were incubated for 12 h with 100 mM 4-thiouridine (4-SU) and then were cross-linked using 365 nm UV light with a CL-1000 Ultraviolet Crosslinker (UVP). After lysis, cell lysates were immunoprecipitated with anti-C/EBPβ or isotypic antibody. RNase T1-treated (final concentration 50 U/ml) and subsequent beads were washed with high-salt wash buffer (50 mM HEPES-KOH, pH 7.5, 500 mM KCl, 0.05% (v/v) NP-40, 0.5 mM DTT, protease inhibitor cocktail (Sigma-Aldrich). For UV-RIP-seq, protein–RNA complex was treated with proteinase K. Immunoprecipitated RNA was purified using acidic phenol, and RNA was subjected to high-throughput sequencing by HiSeq 4000 with PE100 strategy. For RIP-q-PCR analysis, the primers were used listed in Supplementary Table S2. The amount of immunoprecipitated RNAs was represented as the percentile of input RNA (% input).

### Individual-nucleotide resolution cross-linking and IP (iCLIP)

iCLIP was performed. The cells were first subjected to cross-linking with 0.15 J/cm^2^ of 254 nm UV light in a crosslinker HL-2000 (UVP), and then lysed with NP-40 lysis buffer on ice for 10 min and treated with RNAase A (200 ng/ml) for 5 min (Promega). Clear lysates were incubated with anti-C/EBPβ or isotypic antibody overnight at 4 °C. After IP, beads were left for linking biotin-labeled linker. After being separated on a 4%–12% NuPAGE gel (Invitrogen NP0321B0X), the protein–RNA complexes were transferred to NC membrane. Biotin-labeled RNA was detected and visualized according to the instructions of the chemiluminescent kit (Thermo 89880). Protein–RNA complexes were cut from the membrane corresponding to the visualized size of C/EBPβ. RNAs were isolated from the solution with phenol–chloroform and subjected to library construction.

### IP–MASS

IP–MASS was performed according to our previously method^28^. The cells were lysed in IP lysis buffer (Pierce) containing 10% PMSF. Protein A/G magnetic beads (Pierce) were first added into the cell lysates for preclearing. The supernatants were collected after centrifuging at 12,000× *g* rpm and then immunoprecipitated overnight at 4 °C with the indicated antibodies. Protein A/G Magnetic Beads were added into cell lysates and incubated for additional 3 h. After being washed with IP lysis buffer for five times, Protein A/G Magnetic Beads were denatured and resolved by SDS-PAGE gels, and followed by silver staining. The gel lanes containing the immunopurified samples were excised for liquid chromatography–tandem MS analysis by Tsinghua University.

### ChIP-PCR

ChIP-PCR was performed using EZ-ChIP™ Chromatin Immunoprecipitation Kit (Millipore) according to the our previously reported methods^28^. Briefly, the cells were cross-linked with 1% paraformaldehyde and incubated with rotation at room temperature. Cross-linking was stopped after 10 min with glycine to a final concentration of 0.125 M and incubated 5 min further with rotation. Cells were washed with ice-cold PBS (containing 1% PMSF) three times and immediately resuspended in SDS lysis buffer (containing 1% PMSF). Cell lysates were sonicated for 40 cycles of 30 s ON and 30 s OFF in ten cycle increments using a Bioruptor (Diagenode) on ice. After pelleting debris, protein G agarose was added and incubated for 1 h at 4 °C with rotation for preclearing. For IP, precleared cell lysate was incubated with the indicated antibodies overnight with the rotation at 4 °C and protein G agarose was added for the final 2 h of incubation. Beads were washed with low salt, high salt, LiCl wash buffer and chromatin immunocomplex was eluted using elution buffer through incubating at room temperature for 15 min. Reverse crosslinks of protein/DNA complexes to free DNA were realized through adding 5 M NaCl and incubating at 65 °C overnight. qPCR was performed on DNA purified after treatment with RNase (30 min, 37 °C) and proteinase K (2 h, 55 °C) after reversal of crosslinks.

### Statistical analyses

Two-sided Student’s *t* test, one-way ANOVA, and Bonferroni’s Multiple Comparison Test were used to determine significance. These were performed by GraphPad Prism 5 software (GraphPad Software). A 95% confidence interval was considered significant and was defined as *P* < 0.05. *P < 0.05, **P < 0.01, ***P < 0.001.

## Supplementary information

Supplementary Fig. S1-S11; Table S1,S2

## Supplementary information

**Supplementary Information** accompanies the paper at (10.1038/s41421-020-00211-8).
