# Supplementary material for: LncRNA lncLy6C induced by microbiota metabolite butyrate promotes differentiation of Ly6Chigh to Ly6Cint/neg macrophages through lncLy6C/C/EBPβ/Nr4A1 axis
Source: Cell Discov. 2020 Nov 24;6:87. doi: 10.1038/s41421-020-00211-8 (PMC7683537; doi:10.1038/s41421-020-00211-8)
Supplement: Supplementary file 1 — Supplementary Fig. S1-S11; Table S1,S2 [file 41421_2020_211_MOESM1_ESM.pdf]

LncRNA *lncLy6C* induced by microbiota metabolite butyrate promotes differentiation of Ly6C^{high} to Ly6C$^{int/neg}$ macrophages through *lncLy6C* /C/EBPβ/Nr4A1 axis

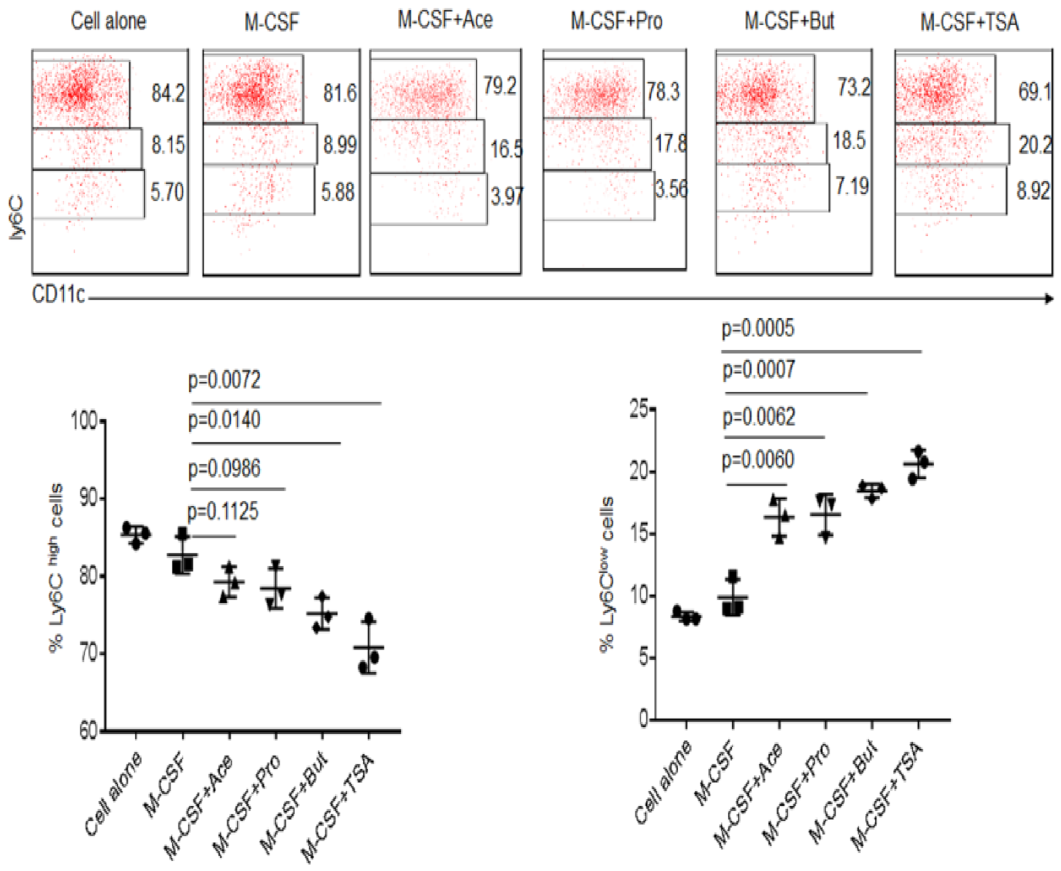

Fig. S1. Flow cytometry of $CD117^-CD11b^+CD115^+$ $Ly6C^+$ BM monocytes after exposed to ascetic acid (Ace, 200 µM), propionic acid (Pro, 200 µM), butyrate (But, 200 µM) and Trichostatin (TSA, 40 nM) for 2 days. % of $Ly6C^{high}$ and $Ly6C^{low}$ cells was compared (lower). Two side Student's *t-test.*

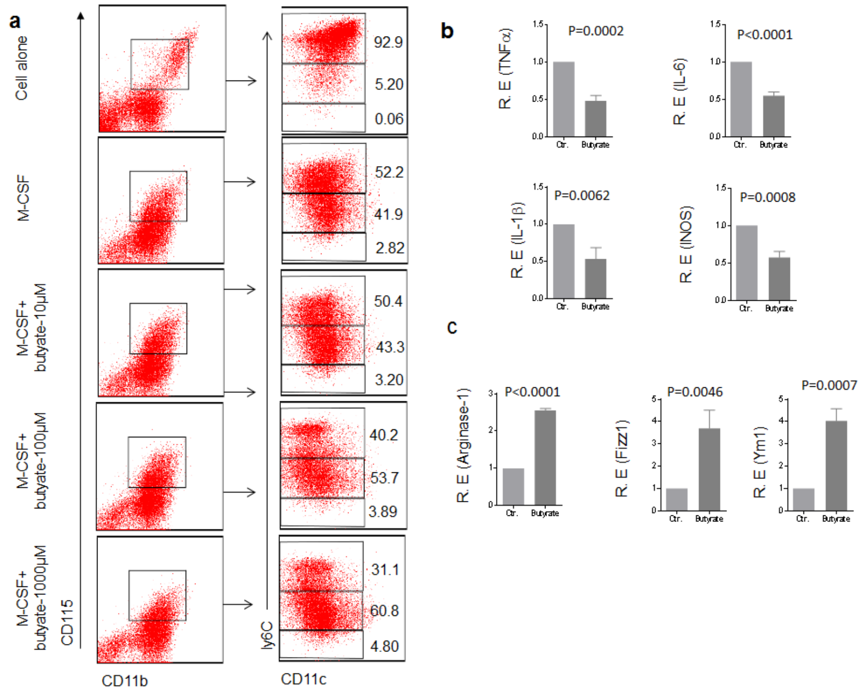

Fig. S2. Charateristics of CD117^{-} CD11b^{+} CD115^{+} Ly6C^{+} BM monocytes after exposed to butyrate. a, Flow cytometry of CD117^{-} CD11b^{+} CD115^{+} Ly6C^{+} BM monocytes after exposed to different concentration (0, 10, 100 and 1000 μM) of butyrate. **b,** QRT-PCR of TNFα, IL-6, IL-1β and iNOS in CD117^{-} CD11b^{+} CD115^{+} Ly6C^{+} BM monocytes after exposed to butyrate (200 μM). Ctr., vehicle. **c**, QRT-PCR of arginase-1, Fizzi1 and Ym1 in CD117^{-} CD11b^{+} CD115^{+} Ly6C^{+} BM monocytes after exposed to butyrate (200 μM). Ctr., vehicle. Two side Student's *t*-test in b and c.

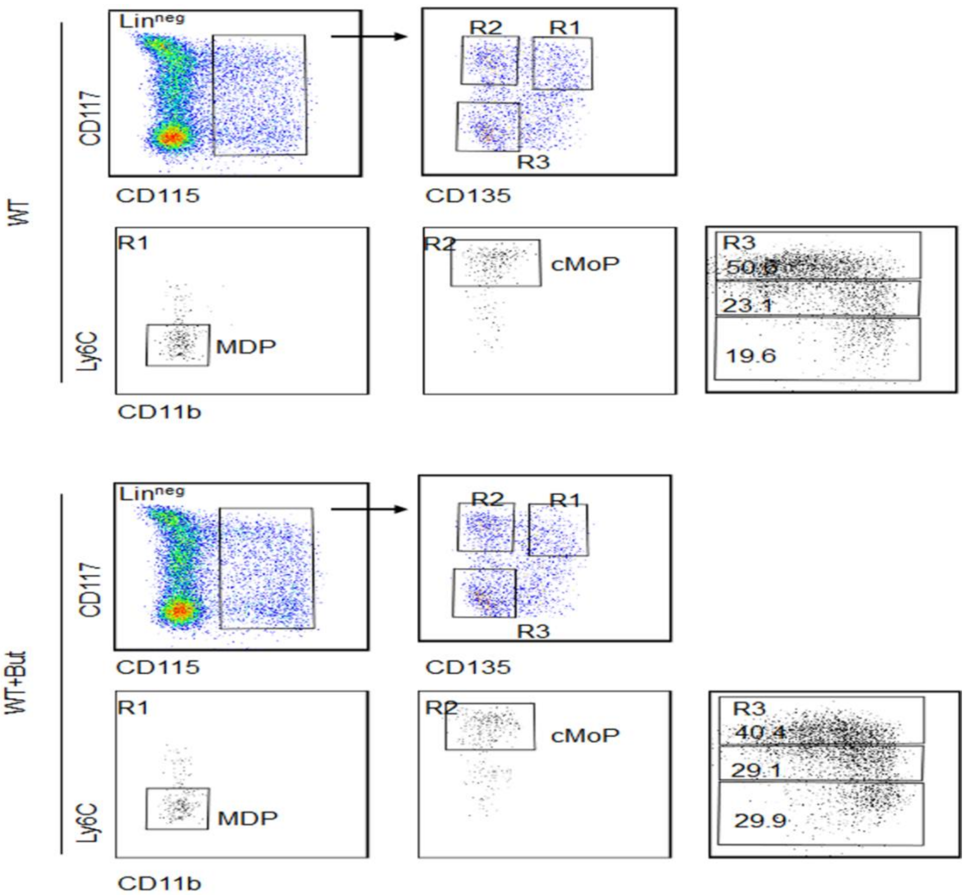

Fig. S3. Flow cytometry of bone marrow cells in wild type mice with (WT+But) or without (WT) butyrate. Mice received sodium butyrate (150 mM) in the drinking water for one week (n=5). One representative of 5 mice.

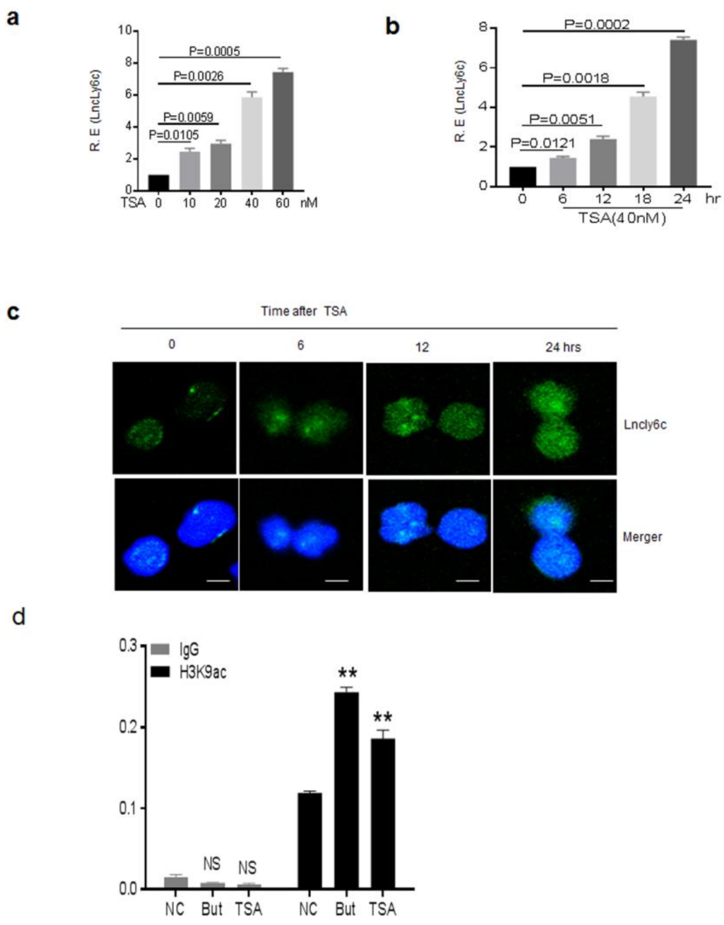

Fig. S4. Trichostatin induces the expression of *lncLy6C*. a, QRT-PCR of *lncLy6C* in BMDMs after exposed to different concentrations of Trichostatin A. R. E, relative expression. **b**, QRT-PCR of *lncLy6C* in BMDMs after exposed to Trichostatin A at different time points. R.E, relative expression. **c**, FISH of *lncLy6C* in BMDMs after exposed to Trichostatin A at different time points. Nuclei were stained with DAPI (blue); Green, lncy6c. Scale bar, 2.5μM. **d**, Chip analyses in BMDMs after exposed to butyrate (200 μM) or TSA (40nM, TSA) for 3 h. Samples were analyzed by ChIP using anti-H3K9Ac. Purified DNA was analyzed by qPCR using primers specific to the promoters of lncly6c. IgG, isotypic antibody. % input was compared. ONE-way ANOVA and Bonferroni's Multiple Comparison Test in a and b.Two side Student's *t-test* in d.

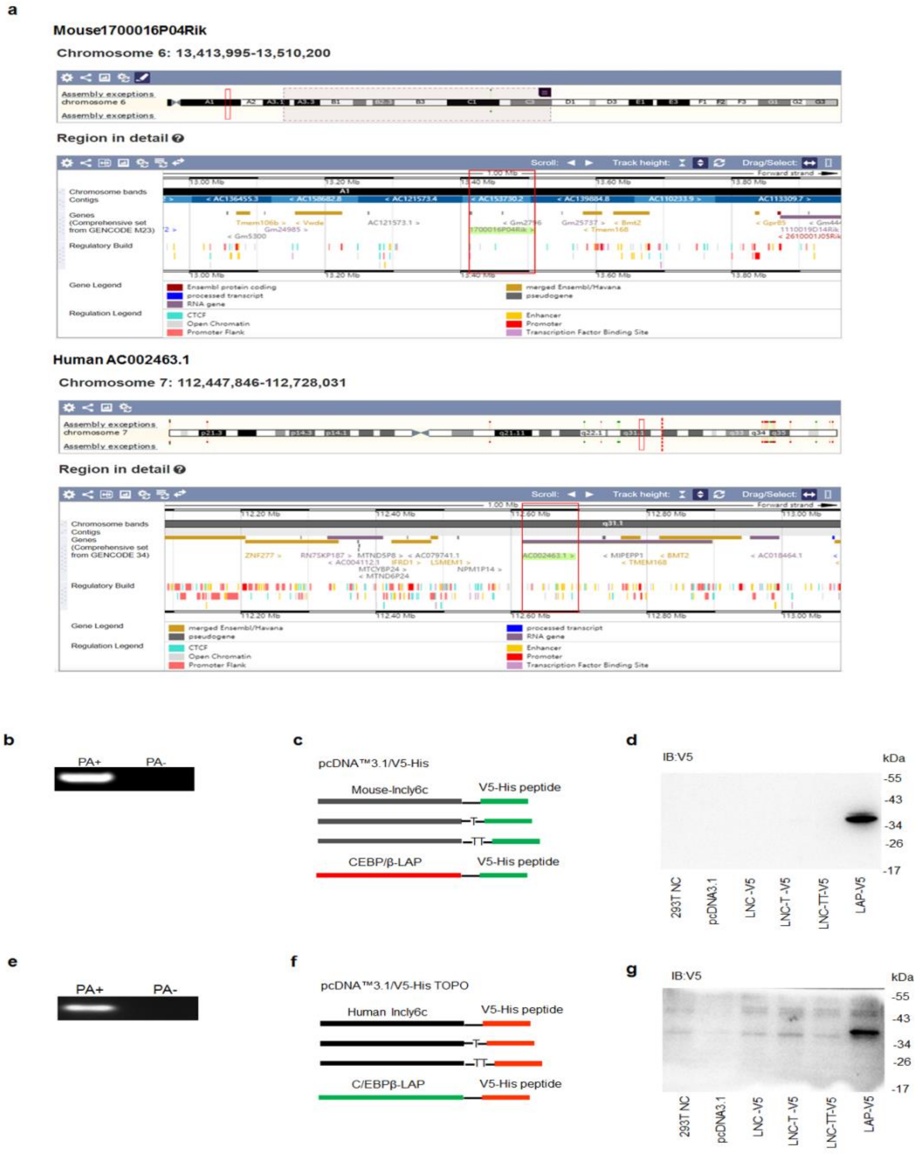

Fig. S5. Characteristics of *LncLy6C*. a, Location of mouse 1700016p04Rik (*lncLy6C*) and human AC0024631 (*Hu lncLy6C*). **b**, RT-PCR assay of mouse *lncLy6C* with polyA+ RNA. The polyA+ RNA fraction of mouse *lncLy6C* was examined using primer with poly-A (PA+) or primers without polyA (PA-).

c and **d**, Immunoblotting of V5-tagged mouse *lncLy6C* transfected BMDMs. Full-length *lncLy6C* was cloned into pcDNA3.1 with N-terminal start codon ATG and C-terminal V5 tag in all three coding patterns (c) , and then these plasmids subsequently were transfected into HEK293T cells separately, and after 48 hours, immunoblotting was used to detect the flag-tagged protein (d). C/EBPβ-LAP with V5 tag, a positive control (d). **e**, RT-PCR assay of hulncLy6C with polyA+ RNA. The polyA+ RNA fraction of hulncly6c was examined using primer with poly-A(PA+) or primers without polyA (PA-). **f** and **g**, Immunoblotting of V5-tagged human *HulncLy6C* transfected BMDMs. Full-length Hu-*lncly6c* was cloned into pcDNA3.1 with N-terminal start codon ATG and C-terminal V5 tag in all three coding patterns (f) , and then these plasmids subsequently were transfected into HEK293T cells

separately, and after 48 hours, immunoblotting was performed using anti -flag antibody (g). C/EBPβ LAP with V5 tag, a positive control. No lncRNA encoding bands (around 20 kd) were found.

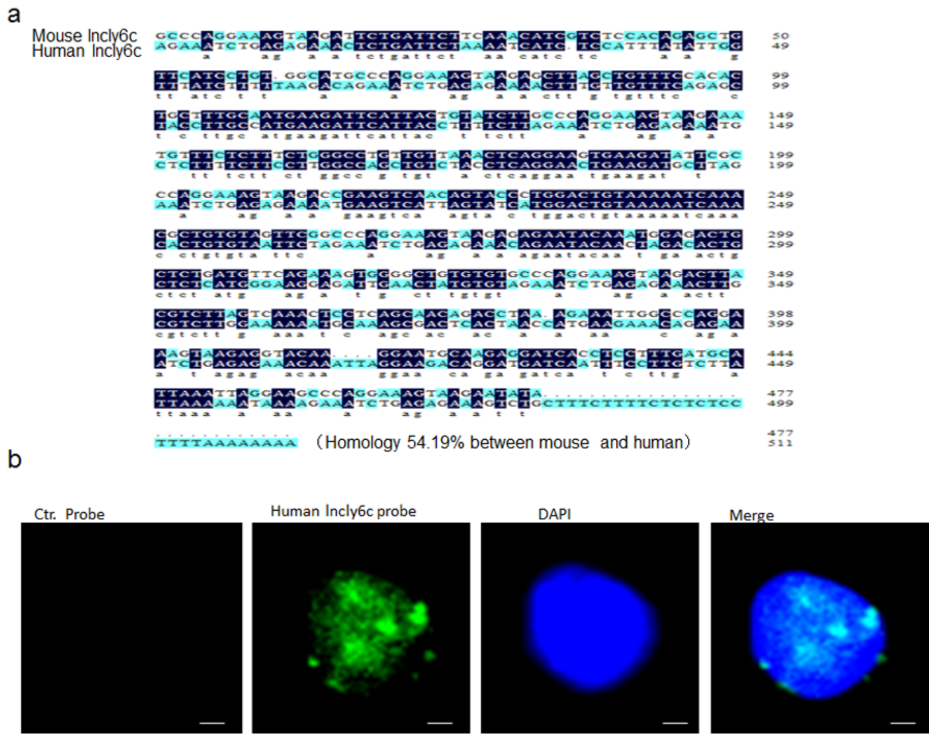

Fig. S6. Homology of human *lncLy6C* with mouse *lncLy6C*.

a, Homology analyses between mouse *lncLy6C* and human *lncLy6C*. Higher homology (54.19%) between mouse *lncLy6C* and human *hulncLy6C* was shown.

b, FISH of *hulncly6c* in $CD14^{+}$ human peripheral blood cells. Green, human lncly6c; Blue, DAPI; Scale bar, 2.5 μM.

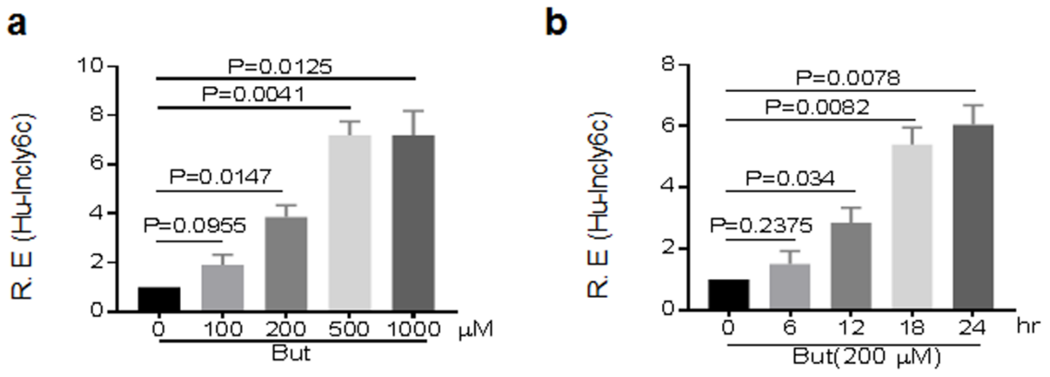

Fig. S7. Expression of *lncLy6C* in human CD14+ monocytes after exposed to butyrate. a QRT-PCR of *lncLy6C* in human CD14+ monocytes after exposed to different concentration of butyrate. R. E, relative expression. **b** QRT-PCR of *lncLy6C* in human CD14+ monocytes after exposed to butyrate (200 μM) at different time points. R. E, relative expression. Two side Student's *t-test.*

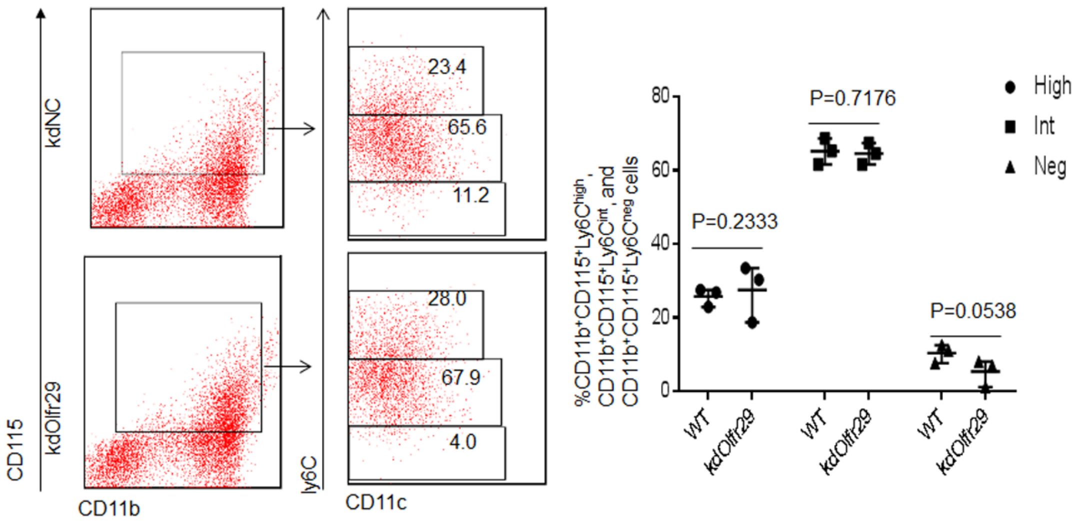

Fig. S8. Flow cytometry of CD11b$^+$CD115$^+$Ly6C^{high}, CD11b$^+$CD115$^+$Ly6C^{int}, CD11b$^+$CD115$^+$Ly6C^{neg} cells in mouse BMC after silencing Olfr29-PS1 (kd Olfr29-PS1). Mouse BMCs were transfected by Olfr29-PS1 siRNA and then cultured for 4 days. KdNC, siRNA control. Two side Student's *t-test.*

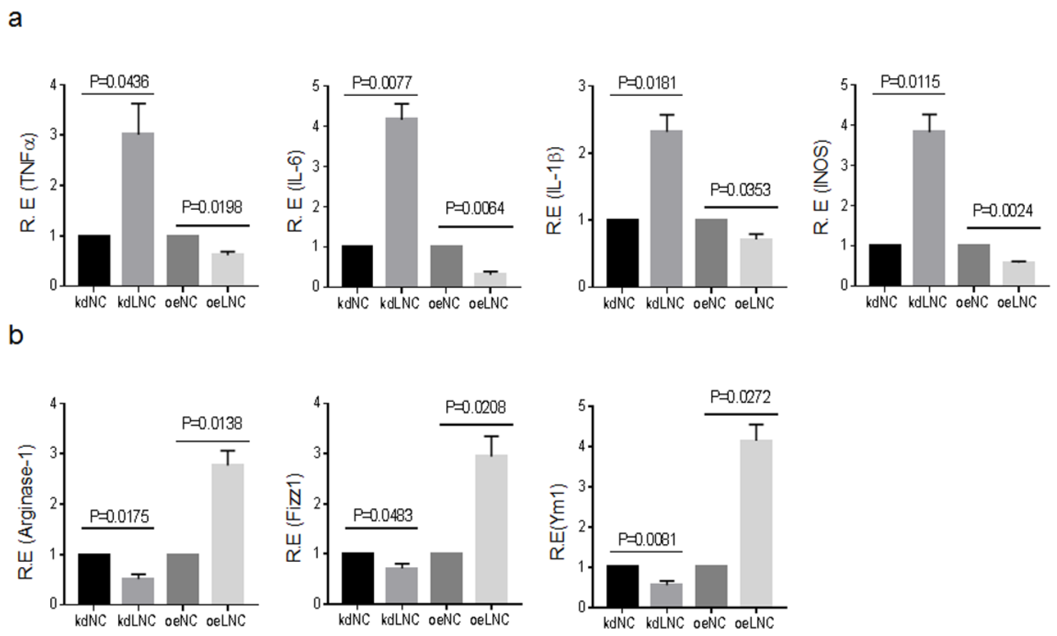

Fig. S9. LncLy6c affects the expression of cytokines, iNOS, Arginase-1, FiZZ1 and Ym1. a QRT-PCR of TNFα, IL-6, IL-1β and iNOS in CD117− CD11b+ CD115+ Ly6C+ BM monocytes after silencing lncly6c or transfecting lncly6c lentiviruses. **b** QRT-PCR of arginase-1, Fizz1 and Ym1 in CD117− CD11b+ CD115+ Ly6C+ BM monocytes after after silencing lncly6c or transfecting lncly6c lentiviruses. kdNC, siRNA control; kdLNC, lncRNA siRNA; OcNC, empty lentivus; OeLNC, LncRNA lentivirus. Two side Student's *t-test.*

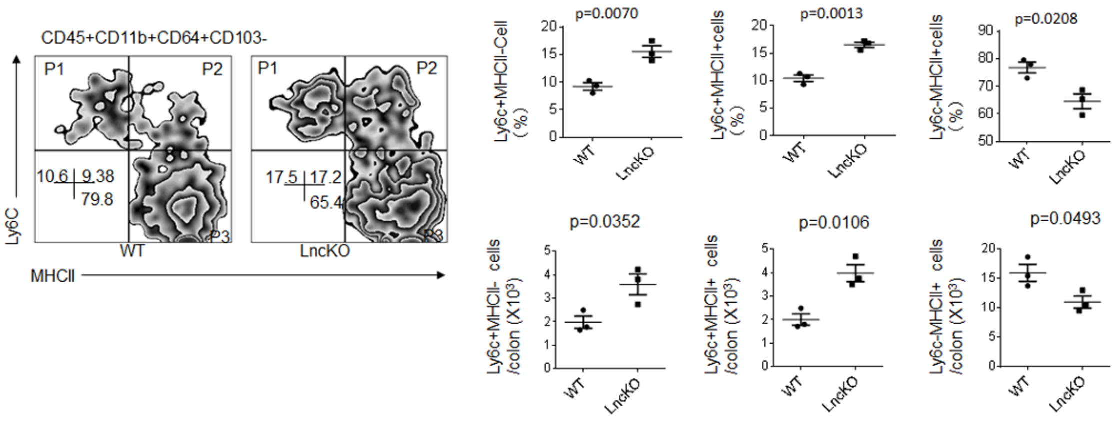

Fig. S10. Flow cytometry of MHCII$^+$Ly6C$^+$ inflammatory macrophages.

CD45$^+$CD11b$^+$CD64$^+$CD103$^-$MHCII$^+$Ly6C$^+$ cells in the colonic tissues of WT or Lncly6c KO mice (n = 6). % cells and total Ly6c$^+$ MHCII$^+$ cell number per colon were analyzed (right). Two side Student's *t-test.*

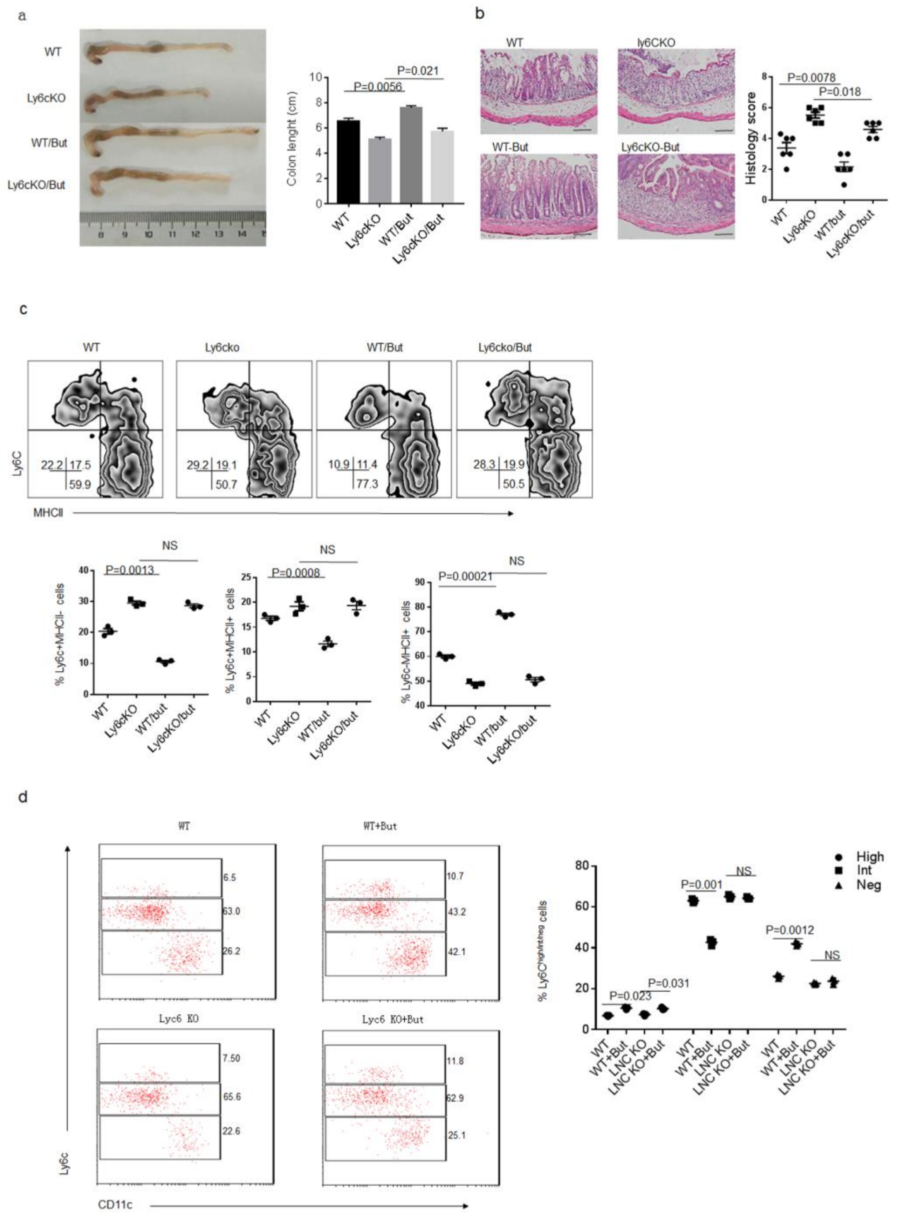

Fig. S11. Butyrate promotes differentiation of Ly6c^{high} inflammatory macrophages into Ly6c^{negative} macrophages in DSS-treated WT but not *Lncly6c* KO mice. a The length of colon in LncLy6c KO and WT mice with or without butyrate after DSS (2.5%) treatment (n=6). **b** H&E staining and histology score of colon tissues in LncLy6c KO and WT mice with or without butyrate after DSS (2.5%) treatment. For histological score, 3 slides/mouse, n=6. Scale bar, 40 μM. **c** Flow cytometry of CD45$^+$CD11b$^+$CD64$^+$CD103$^-$MHC$^+$Ly6C$^+$ in colon tissues in Lynly6c KO and WT mice with or without butyrate after DSS (2.5%) treatment (n=3).

d Flow cytometry of $CD11b^+CD115^+Ly6C^{high}$, $CD11b^+CD115^+Ly6C^{int}$, $CD11b^+CD115^+Ly6C^{neg}$ cells in lncLy6c KO and WT mice with or without butyrate after DSS (2.5%) treatment (n=3). Two side student's *t*-test; NS, no significance.

Supplementary Table S1. Immunoprecipitation in BMDMs using anti-C/EBPβ antibody.

Description	ΣCoverage	Score C/EBPβ Ab	Coverage C/EBPβ Ab	Score Isotypic Ab	Coverage Isotypic Ab	MW [kDa]	calc. pI
C/EBPβ	17.93	8.85	17.93	0	0	15.6	9.95
ASH2L	2.43	2.43	2.43	0	0	60.1	6.95
WDR5	17.07	10.32	17.07	0	0	36.6	8.27
RNF2	2.68	2.68	2.68	0	0	37.6	6.84
CXXC1	15.61	22.72	11.06	7	8	76.1	8.27

Supplementary Table S2. Reagents and oligoes used in this study.

REAGENT or RESOURCE	SOURCE	IDENTIFIER
Antibodies		
β-Actin Antibody	Santa Cruz	Cat:sc-47778 RRID:AB_626632
Anti-C/EBβ Antibody	Abcam	Cat:ab15050 RRID:AB_301598
V5 Tag Monoclonal Antibody	Thermo Fisher Scientific	Cat:MA5-15253 RRID:AB_10977225
Anti-Histone H3 (tri methyl K4) antibody	Abcam	Cat:ab8580 RRID:AB_306649
WDR5 Rabbit mAb	Cell Signaling Technology	Cat:13105 RRID:AB_2620133
Anti-Nr4a1 Antibody	Abcam	Cat:ab13851 RRID:AB_300679
Anti-N6-methyladenosine (m6A) antibody	Abcam	Cat:ab208577 RRID:AB_2753144
Anti-METTL3 antibody	Abcam	Cat:ab195352 RRID:AB_2721254
Anti-STAT3 (phospho Y705) antibody	Abcam	Cat:ab76315 RRID:AB_1658549
Anti-ASH2L antibody	Abcam	Cat:ab50699 RRID:AB_867739
Anti-RbBP5 antibody	Abcam	Cat:ab52084 RRID:AB_882299
Anti-DPY30 antibody	Abcam	Cat:ab126352 RRID:AB_11128034
Anti-MLL1 (D2M7U) Rabbit antibody	Cell Signaling Technology	Cat:14689 RRID:AB_2688009
Rabbit Anti-CD11c antibody	Bioss Inc	Cat: bs-2508R RRID:AB_10855986
Rabbit Anti-CD4 antibody	Bioss Inc	Cat: bs-0766R RRID:AB_ 10857931
Rabbit Anti-CD8 antibody	Bioss Inc	Cat: bs-0648R RRID:AB_ 10857537
Rabbit Anti-CD19 antibody	Bioss Inc	Cat: bs-0079R RRID:AB_ 10857264
Rat Anti-Ly6C Monoclonal antibody	Novus	Cat:NB100-65414 RRID:AB_964346
F4/80 (6A545) antibody	Santa Cruz	Cat:sc-71085 RRID:AB_1122717
APC anti-mouse CD45.2 Antibody	Biolegend	Cat: 109814 RRID:AB_389211
APC anti-mouse CD11c Antibody	Biolegend	Cat: 117310 RRID:AB_ 313779
APC anti-mouse CD117 (c-Kit) Antibody	Biolegend	Cat: 105812 RRID:AB_ 313221
APC/Cy7 anti-mouse/human CD11b Antibody	Biolegend	Cat: 101226 RRID:AB_ 830642
PerCP anti-mouse/human CD11b Antibody	Biolegend	Cat: 135506 RRID:AB_1937253
FITC anti-mouse CD11c Antibody	Biolegend	Cat: 117306 RRID:AB_ 313775
FITC anti-mouse Ly-6C Antibody	Biolegend	Cat: 128006 RRID:AB_ 1186135
FITC anti-mouse CD3 Antibody	Biolegend	Cat: 100204 RRID:AB_312661
FITC anti-mouse CD19 Antibody	Biolegend	Cat: 152404 RRID:AB_2629813
FITC anti-mouse Gr-1 Antibody	Biolegend	Cat: 108406 RRID:AB_313371
PE anti-mouse CD115 (CSF-1R) Antibody	Biolegend	Cat: 135506 RRID:AB_1937253
PE/Cy5 anti-mouse CD135 Antibody	Biolegend	Cat: 135312 RRID:AB_ 2263031
PE/Cy7 anti-mouse Ly-6C Antibody	Biolegend	Cat: 128017 RRID:AB_1732093
Bacterial and Virus Strains		

BL21 Chemically Competent cell	TransGen Biotech	Cat:CD901-01
DH5α Chemically Competent cell	Tiangen Biotech	Cat:CD101-03
Chemicals, Peptides, and Recombinant Proteins		
Recombinant Murine GM-CSF	PeproTech	Cat:315-03
Recombinant Murine M-CSF	PeproTech	Cat:315-02
HiPerFect Transfection Reagent	QIAGEN	Cat:301705
Trichostatin A	MCE	Cat: HY-15144
Pertussis Toxin	MCE	Cat: HY-112779
Polybrene	Millipore	Cat:sc-134220
Lipofectamine™ 3000 Transfection Reagent	Thermo Fisher Scientific	Cat:11668027
Trizol	Life technologies	Cat:15596018
Oligonucleotides for clone genes		
Murine LncLy6C FW	BGI	5’-GGTCGGAGTGGATGGCCC -3’
Murine LncLy6C REV	BGI	5’-TATATTTCCTAATTTAATGCATCAAAGG -3’
Human Lncly6C FW	BGI	5’-GGAGTTGGCTGCCCAGAAATC-3’
Human Lncly6C REV	BGI	5’-TAAAAGGAGAGAGAAAAGAAAGCAG-3’
Murine LncLy6C-L1 FW	BGI	5’-GGATGGCCCAGGAAAGTAAGAC-3’
Murine LncLy6C-L2 FW	BGI	5’-GTAAAAATCAAACGCTGTGTAG-3’
Murine LncLy6C-L3 FW	BGI	5’-GTGTGTCTTACGTCTTAGTC-3’
Murine LncLy6C-L4 FW	BGI	5’-ACAAGGAATGCAAGAGGATC-3’
Murine LncLy6C-MUT1 FW	BGI	5’-CGAAGTCAACAGTACCCTGGGCTGTAAAAATCAAACGCTGTG-3’
Murine LncLy6C-MUT1 REV	BGI	5’-CACAGCGTTTGATTTTTACAGCCCAGGGTACTGTTGACTTCG-3’
Murine LncLy6C-MUT2 FW	BGI	5’-CGAAGTCAACAGTACCCTGGTCTGTAAAAATCAAACGCTGTG-3’
Murine LncLy6C-MUT2 REV	BGI	5’-CACAGCGTTTGATTTTTACAGACCAGGGTACTGTTGACTTCG-3’
Murine LncLy6C-MUT3 FW	BGI	5’-CGAAGTCAACAGTACCCTGGCTGTAAAAATCAAACGCTGTG-3’
Murine LncLy6C-MUT3 REV	BGI	5’-CACAGCGTTTGATTTTTACAGCCAGGGTACTGTTGACTTCG-3’
Murine LncLy6C-MUT4 FW	BGI	5’-AGTTCGGAGAATACAAATGGGGACTGCTCTGATGTTCAGAAAG-3’
Murine LncLy6C-MUT4 REV	BGI	5’-CTTTCTGAACATCAGAGCAGTCCCCATTTGTATTCTCCGAACT-3’
Murine LAP* FW	BGI	5’-GCCACCATGCACCGCCTGCTGGCCTGGGACG-3’
Murine LAP* REV	BGI	5’-GCAGTGGCCCGCCGAGGCCA-3’

Murine LAP FW	BGI	5'-GCCACCATGGAAGTGGCCAACTTCTACTACGAG C-3'
Murine LAP REV	BGI	5'-GCAGTGGCCCGCCGAGGCCA-3'
Murine LIP FW	BGI	5'-GCCACCATGGCGGCCGGTTTCCCGTTCG-3'
Murine LIP REV	BGI	5'-GCAGTGGCCCGCCGAGGCCA-3'
Murine ΔLAP* FW	BGI	5'-GCCACCATGCACCGCCTGCTGGCCTGGGACG-3'
Murine ΔLAP* REV	BGI	5'-GGCGGGCGCGTCGTCCGCGCGCTTG-3'
Murine ΔLAP FW	BGI	5'-GCCACCATGGAAGTGGCCAACTTCTACTACGAG C-3'
Murine ΔLAP REV	BGI	5'-GGCGGGCGCGTCGTCCGCGCGCTTG-3'
Murine WDR5 FW	BGI	5'-GCCACCATGGCCACAGAGGAGAAGAAGC-3'
Murine WDR5 REV	BGI	5'-GCAGTCACTCTTCCACAGTTTGATT-3'
Murine ASH2L FW	BGI	5'-GCCACCATGGCGGCGGCTGGAGCGGGT -3'
Murine ASH2L REV	BGI	5'-GGGTTCCCAGGGTGGACTACGTCTT-3'
Murine DPY30 FW	BGI	5'-GCCACCATGGAGTCGGAGCAGATGCTGGAGG-3'
Murine DPY30 REV	BGI	5'-ATTTCGATCTTCAAACTGCGCCTTG-3'
Murine RBBP5 FW	BGI	5'-GCCACCATGAACCTCGAGTTGCTGGAGTCCT-3'
Murine RBBP5 REV	BGI	5'-CAGCAGTTCTGAGATGGCTCCTCCT-3'
Oligonucleotides for qRT-PCR		
Murine GAPDH FW	BGI	5'-TCAACGGCACAGTCAAGG-3'
Murine GAPDH REV	BGI	5'-TACTCAGCACCGGCCTCA-3'
Murine Arg1 FW	BGI	5'- CTGACCTATGTGTCATTTGGG -3'
Murine Arg1 REV	BGI	5'- TCAGGAGAAAGGACACAGGTT -3'
Murine INOS FW	BGI	5'-CTTGGAGCGAGTTGTGGATTGT-3'
Murine INOS REV	BGI	5'-AGGTGAGGGCTTGGCTGAGTGA-3'
Murine TNFa FW	BGI	5'-CCAGACCCTCACACTCAGATCA-3'
Murine TNFa Rev	BGI	5'-GTAGACAAGGTACAACCCATCG-3'
Murine IL-1b FW	BGI	5'-GCAGGCAGTATCACTCATTGTG-3'
Murine IL-1b Rev	BGI	5'-AGGCTTTTTTGTTGTTCATCTC-3'
Murine IL-6 FW	BGI	5'-ACAACCACGGCCTTCCCTACTT-3'
Murine IL-6 Rev	BGI	5'-TTTCTCATTTCCACGATTTCCC-3'
Murine FiZZ1 FW	BGI	5'-AGACTACAACTTGTTCCCTTCT-3'
Murine FiZZ1 Rev	BGI	5'-GTTCCTTGACCTTATTCTCCAC-3'
Murine YM-1 Fw	BGI	5'-CGTAATCAAGTCTGGGTACAAG-3'
Murine YM-1 Rev	BGI	5'-AGGGTCACTCAGGATAAAGGTA-3'
Murine Nr4A1 Fw	BGI	5'-TGAGTTCGGCAAGCCTACCAT-3'
Murine Nr4A1 Rev	BGI	5'-GGAGGAGGCAGAGGAACAAGC-3'
Murine LncLy6C FW	BGI	5'-CCCAGGAAAGTAAGACCA-3'
Murine LncLy6C REV	BGI	5'-TTTAACAACAGGCCCAGA-3'
Human Lncly6C FW	BGI	5'- AGACATGTAAAGTCCTGCTCACC-3'
Human Lncly6C REV	BGI	5'- CTTCATAAGCATCTTCAGTTCCT-3'
Murine Gm8883 FW	BGI	5'-GTCTTGTGTGTGCTAGCTCTCT-3'
Murine Gm8883 REV	BGI	5'-CTGTCCTGGAACTCACTCTGTA-3'
Murine 5730403I07Rik FW	BGI	5'- AAGGGAACTTCACGGTGGT -3'

Murine 5730403I07Rik REV	BGI	5'- TGATGTTTCAGCGAGGGA-3'
Murine Efcab8 FW	BGI	5'-TGTGCCTACTGCTCTTCTTCCC-3'
Murine Efcab8 REV	BGI	5'-TGCCTCCTTCTCAAACACCTTC-3'
Murine Meg3 FW	BGI	5'-TTGAAATAAAGGAGTGACAGAA -3'
Murine Meg3 REV	BGI	5'-CCCAGAATAAGATGAGCAGAAG-3'
Murine Olfr29-ps1 FW	BGI	5'-GACATCTGCTACACCACCTCCT-3'
Murine Olfr29-ps1 REV	BGI	5'-CGATCAAACGCCATCATACTCA-3'
Murine 4930528A17Rik FW	BGI	5'-AGTTCTGGAAGCAGGGTGTTTG-3'
Murine 4930528A17Rik REV	BGI	5'-CTTTTACCTCCTTTCGGGAGGC-3'
Murine BC021767 FW	BGI	5'-AGCAGGGAGGGAACATTATGGG -3'
Murine BC021767 REV	BGI	5'-TGAGAGTAAAGCAGCAAGGCAG -3'
Murine 1500009C09Rik FW	BGI	5'-TTCTGTTCCCATCTCTGTTGC-3'
Murine 1500009C09Rik REV	BGI	5'-GGTGAGCGGGGCTGATCTATA-3'
Murine Gm5468 FW	BGI	5'-TTGCTTGTTCTGCTTCGTCCT-3'
Murine Gm5468 REV	BGI	5'-CCTTTGGCATTCGTCTCCCTA-3'
Murine Gm5105 FW	BGI	5'-CAGAGAGCGTGGTAGAGGATT-3'
Murine Gm5105 REV	BGI	5'-GGAAGAGACAAAGGGGGACAT-3'
Murine Speer5-ps1 FW	BGI	5'-ACAAGCAAGAAGCAGTTTGAG-3'
Murine Speer5-ps1 REV	BGI	5'-GCTGGGCAGGTTACGATAGAA-3'
Oligonucleotides used in 5'- and 3'-RACE		
Murine LncLy6C 5'RACE	BGI	5'-CAATTTCTTTAGGTCTGTTGCT -3'
Murine LncLy6C 3'RACE	BGI	5'-ATCTTAATGTTTCTCTTTCTGG -3'
UPM long primer for 5'RACE	BGI	5'-TAATACGACTCACTATAGGGCAAGCAGTGGTATCAACGCAGAGT-3'
UPM short primer for 5'RACE	BGI	5'-CTAATACGACTCACTATAGGGC-3'
Oligonucleotides used in CHIP-RCR		
Nr4A1-H3K4me3 FW for CHIP-PCR	BGI	5'-CCTCCTCCTGGCCGCCTCCC-3'
Nr4A1-H3K4me3 REV for CHIP-PCR	BGI	5'-AGCACACTCCCCCAACTTTC-3'
Lncly6C-H3K9ac FW for CHIP-PCR	BGI	5'-GCCCAGGAAAGTAAGACCATAGC-3'
Lncly6C-H3K9ac REV for CHIP-PCR	BGI	5'-ACAACAGGCCCAGAAAGAGAAAC-3'
C/EBPβ FW for CHIP-PCR	BGI	5'- TCCACAAACAGAAAGCCTACCT -3'
C/EBPβ REV for CHIP-PCR	BGI	5'- ACCTTCCCAGTGTCAACCCAAT -3'
Oligonucleotides used in Northern blot		
T7- Murine LncLy6C -FW	BGI	5'- CTGATTCTTCAAACATCGTCTC-3'
T7- Murine LncLy6C-REV	BGI	5'-TAATACGACTCACTATAGGGCATTCCTTGTACCCAATTTCTT -3'
T7-U6 RNA-FW	BGI	5'-GTGCTCGCTTCGGCAGCACATATAC-3'
T7-U6 RNA-REV	BGI	5'-TAATACGACTCACTATAGGGAAAAATATGGAACGCTTCACGAATT-3'
Probes used in the RNA-FISH		
Murine LncLy6C-FAM	BGI	5'-FAM-AAGCATCTTCAGTTCCTGAGGTAGA -3'
Human LncLy6C-FAM	BGI	5'- FAM-ATTTTTTCCAAGACGCAAGACACAT-3'

NC-FAM	BGI	5’-FAM--CGGGAGCCTAGGAAGTGCATCTTTC-3’
siRNAs used in this study		
Murine LncLy6C	Ribobio	5’-CCTCAGCAACAGACCTAAA-3’
Mettl3 siRNA-1	Ribobio	5’-GCTACCGTATGGGACATTA -3’
Mettl3 siRNA-2	Ribobio	5’-CCGCAAGATTGAGTTATTT -3’
Other		
pcDNA™3.1/V5-His TOPO® TA Expression Kit	Invitrogen	Cat: K4800-40
Pierce™ Magnetic RNA-Protein Pull-Down Kit	Thermo Fisher Scientific	Cat:20164
Dual-Luciferase® Reporter Assay System	Promega	Cat: E1960
DIG Northern Starter Kit	Roche	Cat:12039672910
Pierce™ Protein G Agarose	Thermo Fisher Scientific	Cat: 20397
FirstChoice RLM-RACE Kit	Ambion	Cat: AM1700
EZ-ChIP™Chromatin Immunoprecipitation Kit	Millipore	Cat:17-371
